# Polygenic Risk Scores disclosure for cardiovascular prevention: Protocol of the Personalized HeartCare (PHC) trial

**DOI:** 10.1371/journal.pone.0345294

**Published:** 2026-04-06

**Authors:** Luigi Russo, Luca Proto, Sara Farina, Eleonora Pascucci, Riccardo Galarducci, Tina Pasciuto, Anna Severino, Giovanna Liuzzo, Andrea Urbani, Ivo Iavicoli, Roberta Pastorino, Stefania Boccia

**Affiliations:** 1 Section of Hygiene, Department of Life Sciences and Public Health, Università Cattolica del Sacro Cuore, Rome, Italy; 2 Research Core Facilty Data Collection G-SteP, Fondazione Policlinico Universitario Agostino Gemelli IRCCS, Rome, Italy; 3 Department of Cardiovascular and Pulmonary Sciences, Università Cattolica School of Medicine, Rome, Italy; 4 Department of Cardiovascular Science, Fondazione Policlinico Gemelli - IRCCS, Rome, Italy; 5 Department of Diagnostic and Laboratory Medicine, Unity of Chemistry, Biochemistry and Clinical Molecular Biology, Fondazione Policlinico Universitario A. Gemelli IRCCS, Rome, Italy; 6 Section of Occupational Medicine, Department of Healthcare Surveillance and Bioethics, Fondazione Policlinico Universitario A. Gemelli IRCCS, Rome, Italy; 7 Department of Woman and Child Health, Fondazione Policlinico Universitario A. Gemelli IRCCS, Rome, Italy; PLOS: Public Library of Science, UNITED KINGDOM OF GREAT BRITAIN AND NORTHERN IRELAND

## Abstract

**Background:**

Cardiovascular diseases (CVDs) are the leading cause of death and morbidity in Europe, highlighting the need for innovative and personalized prevention strategies. Communicating genetic risk through Polygenic Risk Scores (PRSs) may help promote healthier behaviors, though evidence remains limited. This trial aims to assess whether PRS disclosure can support lifestyle changes and its implementation into clinical practice.

**Methods:**

The PHC study is a single-arm clinical trial whose primary objective is to assess the effectiveness of communicating CVD PRSs in modifying the lifestyle behaviors, based on the Life’s Essential 8 (LE8) questionnaire. Secondary endpoints include changes in SCORE2 and LDL-cholesterol levels. Additionally, the study aims to evaluate the feasibility and acceptability of the intervention for participants and physicians, and the working ability through the Work Ability Index. The trial enrolls 650 healthy participants from the general population, aged over 40 years, who must have a 10-year CVD risk of less than 10% on the SCORE2 charts or less than 15% on the SCORE2-OP. All participants undergo an initial assessment at baseline (T0), a disclosure visit one month later (T1) to receive the PRS and lifestyle results, and health recommendations, and a final follow-up at six months (T2) for the reassessment of lifestyle behaviors and modifications.

**Discussion:**

This study explores the innovative use of PRS to enhance primary prevention of cardiovascular diseases, potentially improving individual risk stratification beyond traditional clinical models. By combining PRS with conventional factors, it aims to test whether disclosure can motivate lifestyle improvements. Furthermore, it explores the practical integration of PRS communication into routine care, assessing both participant acceptance and clinician workflow impact, offering insights into real-world implementation of PRS. The findings may inform how PRS can be integrated into clinical workflows, contributing to advance personalized prevention strategies, and help implement PRS-based interventions in health systems.

**Trial registration number:**

ClinicalTrials.gov NCT06888466.

## Introduction

Cardiovascular diseases (CVDs) are the leading cause of mortality and morbidity in Europe, posing a threat to the sustainability of healthcare systems [1]. Their impact on population health is progressively increasing, both in terms of years lost due to disability and years lived with a lower quality of life [2]. CVDs have a multifactorial etiology, arising from the complex interplay of various determinants, including unhealthy lifestyle behaviors, environmental and social factors, and genetic predisposition [3,4]. This complexity necessitates comprehensive, large-scale primary prevention strategies, the design and implementation of which pose significant challenges [5].

It is well demonstrated that improving dietary habits toward healthier diets, increasing physical activity, and reducing or preferably discounting tobacco use can be highly effective [6]. In this context, Polygenic Risk Scores (PRSs), which aggregate the effects of genetic variants identified through genome-wide association studies (GWAS), offer an opportunity to improve CVD preventive strategies and personalized care. PRS is able to quantify genetic predisposition to a phenotype, identifying individuals at elevated risk for major cardiovascular events like myocardial infarction and stroke [7,8], and represent a non-modifiable risk factor for CVDs, providing an estimate of an individual’s lifetime risk of developing such conditions. Previous validation studies like GENVASC in the UK or CV-GENES in Brazil, have shown that integrating PRS with traditional risk models have the ability to identify high-risk individuals, who could benefit from early interventions, but also can improves predictive accuracy, thus enhance risk stratification [9–12].

PRS has demonstrated improved predictive accuracy for several cardiometabolic diseases, including coronary artery disease (CAD), atrial fibrillation (AF), and type 2 diabetes mellitus (T2DM), especially when integrated into established clinical risk models [13]. In addition, ancestry-specific PRSs have been validated for CAD and atherosclerotic cardiovascular disease (ASCVD), showing significant improvements in risk stratification and net reclassification [13]. While genetic predisposition cannot be altered, its clinical manifestation may be mitigated through behavioral and therapeutic interventions, reinforcing the value of PRS in supporting personalized prevention strategies [14]. Communicating genetic risk may enhance individuals’ awareness and motivate positive behavioral changes, such as adopting healthier diets, increasing physical activity, quitting smoking, and better managing modifiable risk factors like low-density lipoprotein cholesterol and blood glucose levels [13,15]. The American Heart Association has highlighted that individuals with high CAD PRS may experience greater absolute benefit from lifestyle and pharmacological interventions, such as statins and PCSK9 inhibitors, compared to those with lower genetic risk [13]. Recent studies suggest that PRS disclosure can positively influence health-related behaviors [15,16]; however, conclusive evidence on its clinical efficacy remains limited. Moreover, data on the broader impact of PRS communication on patients, healthcare providers, and the healthcare system are still lacking [17,18]. A thorough and timely evaluation of these dimensions is essential to support the effective integration of PRS into routine clinical practice.

The implementation of PRS in preventive strategies aligns with broader efforts to modernize healthcare through personalized and innovative approaches [16]. In line with these goals, the Digital Lifelong Prevention (DARE) project has been launched within the framework of the European Next Generation EU initiative and the Italian National Recovery and Resilience Plan (PNRR) [17]. With the overarching aim of fostering healthcare innovation, strengthening disease prevention, and improving early diagnosis, DARE supports a broad range of pilot studies designed to test novel approaches in public health and precision medicine.

As part of the DARE initiative, facilitating the integration of PRS into clinical practice is particularly relevant, and the present study is positioned within this framework. This protocol describes a single arm clinical trial aimed at evaluating the effectiveness of disclosing cardiovascular genetic risk (PRS) in promoting the adoption of healthier lifestyle behaviors. Additionally, the study aims to assess the acceptability of the intervention, participants’ and healthcare professionals’ understanding of PRS, and the potential implications in terms of healthcare staff workload.

## Methods

This research protocol was developed following SPIRIT (Standard Protocol Items: Recommendations for Interventional Trials) statement [18,19].

### Hypothesis

The hypothesis is that participants receiving the disclosure of their PRS combined with the personalized coaching on risk factors will demonstrate significant improvements in lifestyle behaviors compared to their baseline measurements.

### Objective

#### Primary objective.

The primary objective of the study is to assess the effectiveness of communicating the PRS in modifying participants’ lifestyle, measured through the Life's Essential 8 questionnaire [20].

#### Secondary objective.

The secondary objective is to evaluate the feasibility of integrating the PRS into the participants care pathway, and to examinechanges in the WAI.

### Study design

**Fig 1:**
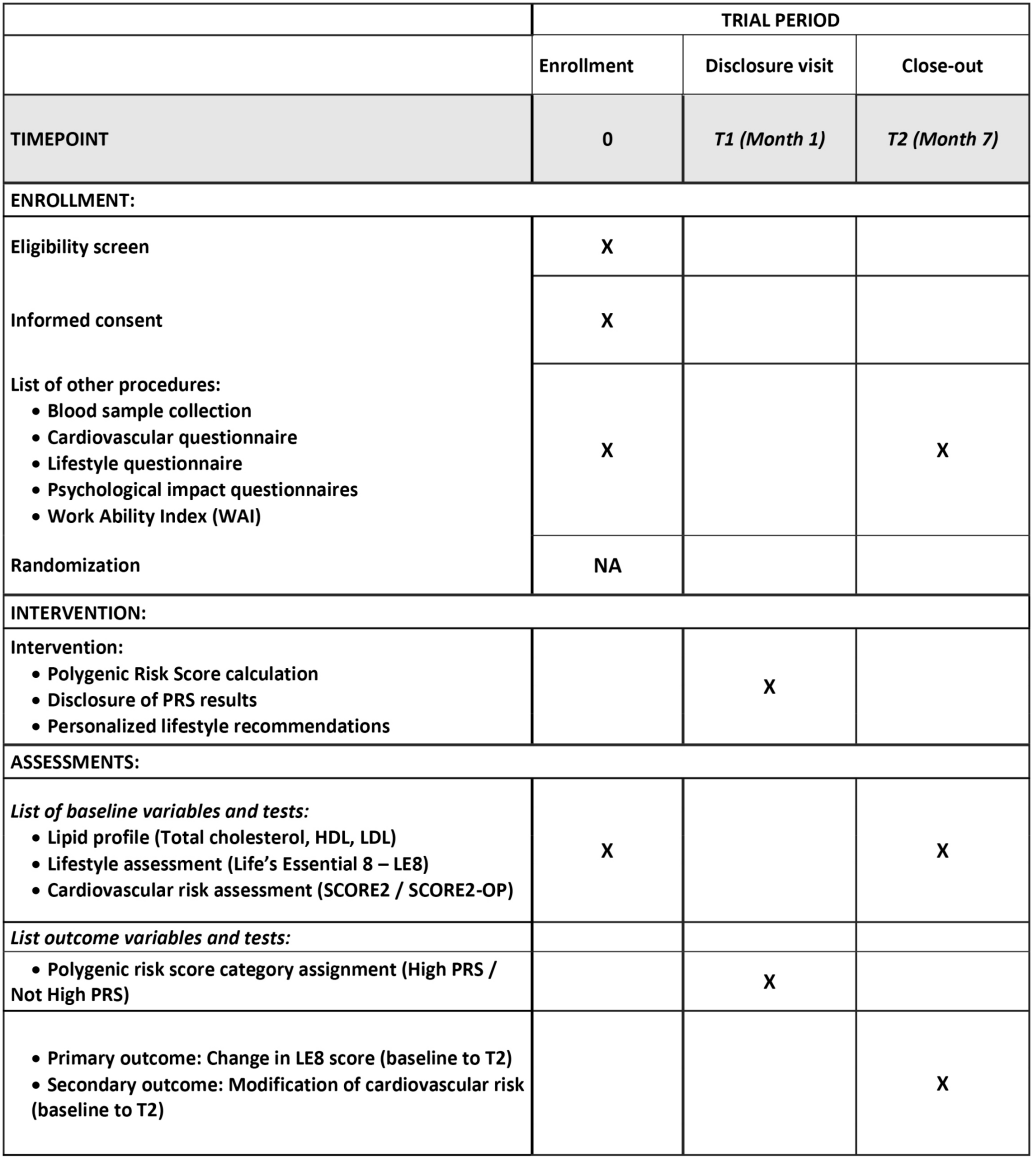
Participant timeline: Schedule of enrollment, interventions, and assessments. PRS = Polygenic Risk Score LE8 = Life’s Essential 8; HDL = High-Density Lipoprotein Cholesterol; LDL = Low-Density Lipoprotein Cholesterol.

This is a monocentric, non-randomized, single-arm pre-post clinical trial (Fig 1).

### Participants, study setting and recruitment

The target population consists of healthy general population. Recruitment will take place at the cardiovascular prevention outpatient clinics.

The recruitment process will span a period of 6 months, and each participant will be followed for a total of 7 months.

The first participant was enrolled in June 2025, and enrollment will continue until the end of March 2026. Completion of the final follow-up is anticipated by December 2026. Data analysis and dissemination of results are expected to be completed by March 2027.

### Eligibility criteria

The study will include individuals older than 40 years old, who do not have established CVDs, diabetes mellitus, or familial hypercholesterolemia.

Additionally, participants must have a 10-year CVD risk of less than 10% according to the SCORE2 charts [21] or less than 15% according to the SCORE2-Older Persons (SCORE2-OP) charts [22]. Participants with a very high 10-year CVD risk (SCORE2 ≥ 10% or SCORE2-OP ≥ 15%) will be excluded from the study.

### Intervention

Participants undergo an intervention that consists in assessing their genetic predisposition to CVDs trough the determination of PRS. As such, following enrollment, a blood sample is drawn from each participant for DNA extraction and analysis. Participants will be classified into two levels of PRS for CVD based on the results: high PRS, and not-high PRS. Participants will receive personalized in-person consultation with a medical cardiologist to obtain tailored guidance on CVD prevention, based on the PRS results and traditional risk factors, according to the ESC guidelines [23].

### Study procedure

#### Enrollment (T0).

Participants will be enrolled during the initial visit (T0). After providing written informed consent (S1 File), they will undergo the baseline assessment, including the collection of demographics, socioeconomic, work ability index (WAI) [24] and clinical data, as well as an evaluation of their lifestyle using the Life’s Essential 8 (LE8) questionnaire. The physician will conduct a medical examination to record biometric parameters (body mass index (BMI), body circumference, heart rate, and blood pressure). Participants will also provide recent blood test results (maximum 6 months ahead of the visit), including lipid profile (LDL-cholesterol, HDL-cholesterol, triglycerides), glucose, or hemoglobin A1c. For those not able to provide recent analysis results, a new determination will be done. Additionally, a blood sample will be collected for PRS analysis. Participants will also complete questionnaires regarding their knowledge, perceptions, and attitudes toward cardiovascular health and PRS.

#### Disclosure visit (T1).

One month after T0, participants will attend an informative visit (T1) during which the physician will disclose their PRS results, their genetic risk category and lifestyle score. Participants will receive personalized recommendations aimed at promoting healthier behaviors, based on the combination of the genetic and lifestyle measure.

#### Follow-up visit (T2).

Six months after T1, a final evaluation (T2) is conducted to monitor changes in lifestyle. Participants will complete again the LE’8 questionnaire, and will undergo biometric measurements, including weight, BMI, body circumference, heart rate, and blood pressure. A blood sample will be drawn for evaluation of modification in the lipid profile (LDL-cholesterol, HDL-cholesterol, triglycerides), glucose, or hemoglobin A1c. Furthermore, we will dose lipoprotein (a) and troponin I, and administer the WAI.

### Questionnaire(s)

Various questionnaires will be administered during the course of the study, to evaluate lifestyle category, psychological outcomes and feasibility and acceptability of the intervention. Questionnaires are reported in Table 1, while the whole questionnaire is available as S2 File.

**Table 1:** Administered questionnaires.

Questionnaire	Domain	Characteristics	Time point(s)
Life’s Essential 8 (LE8) [20,25]	Lifestyle category	It assesses the cardiovascular health (CVH) on a 0–100 scale. 0–49: poor CVH; 50–79: intermediate CVH; 80–100: optimal CVH	T0, T2
GAD-7 [26]	Anxiety	Score from 0 to 21. Anxiety levels: minimal (0–4), mild (5–9), moderate (10–14), and severe (15–21)	T0, T2
Vanderbilt PRS-KS [27]	Knowledge of PRS	Seven-item questionnaire with dichotomous “true” or “false” responses.	T0
HBCVD [28]	Knowledge of CVD (awareness and prevention)	25 items on 1–4 Likert scale. Low, moderate, or high knowledge levels.	T0
Feeling About Genomic Testing Results (FACToR) [29,30]	Psychological impact of genomic test disclosure	12-item questionnaire. Four domains assessed: negative emotions, positive emotions, uncertainty, and privacy concerns	T1
Physicians’ perceptions [31–33]	Feasibility and acceptability of the intervention	11-item questionnaire on a 1–4 Likert scale.	T2
Participants’ perceptions [31–33]	Acceptability of the intervention	7-item questionnaire on a 1–4 Likert scale.	T2
Work Ability Index (WAI) [24,34]	Work ability	Sixty items distributed in seven subscales.	T0, T2

### Outcomes

#### Primary outcome.

Change in the lifestyle profile of the participants.

#### Secondary outcomes.

1. Change in the LDL-Cholesterol levels of the participants.
2. Change in the SCORE-2 of the participants.
3. Behavioral changes related to health, including smoking and dietary habits, and physical activity.
4. Psychological impact of testing.
5. Acceptability of the PRS testing for participants and clinicians.
6. Feasibility of the integration of PRS testing into care pathway.
7. Change in the work ability of participants.

### Endpoints

#### Primary endpoint.

The primary endpoint will be the difference in lifestyle score between baseline and final follow-up, measured using the LE8 tool. The LE8 score is calculated on a continuous scale ranging from 0 to 100, with higher scores indicating better CVH. An improvement of at least 3 points will be considered successful [35].

#### Secondary endpoints.

1. Change in the LDL-Cholesterol levels between baseline and final follow-up.
2. Change in the SCORE-2 between baseline and final follow-up.
3. Percentage of participants who quit smoking or reduced their smoking habits at final follow-up.
4. Percentage of participants who improved their dietary patterns at final follow-up.
5. Number of participants who increased their physical activity levels at final follow-up.
6. Change in anxiety levels between baseline and final follow-up.
7. Proportion of clinicians reporting high acceptability of PRS testing, measured through a structured questionnaire.
8. Proportion of clinicians reporting high feasibility of implementing PRS testing in routine clinical practice.
9. Change in the self-assessment of the working ability among working individuals.

### Laboratory procedures

#### Sample processing.

After collection, blood samples intended for PRS analysis will be stored in 6 mL EDTA tubes (K3E K3ESTA) to ensure DNA stability, and then aliquoted into the laboratory in 2 mL tubes for DNA extraction. The remaining blood volume will be temporarily stored at −20°C and subsequently discarded after PRS analysis is completed. DNA extraction will be performed using the KingFisher Duo Prime automated extractor in combination with the MagMAX DNA Multi-Sample Ultra 2.0 Kit, ensuring high yield and purity. The extracted DNA will then undergo quantification and quality assessment via spectrophotometry, while its integrity will be evaluated through gel electrophoresis and then considered suitable.

#### Biological analyses.

DNA samples will be processed for genotyping using microarray technology on the GeneTitan MC Fast Scan Instrument, with the Axiom Precision Medicine Diversity Array Plus Kit, 96-format (PMDA).

The selection of genotyped SNPs for inclusion in the score is based on an existing, validated PRS based on a European population, from the study by Khera et al [36]. For this study, the PRS used is the one developed by Khera et al. includes 6,630,150 single nucleotide polymorphisms (SNPs) associated with coronary artery disease (CAD).

Quality control (QC) procedures, including the removal of SNPs with a minor allele frequency (MAF) below 1% and those failing the Hardy-Weinberg equilibrium (HWE) test with a P-value < 10−10, will be performed to ensure the reliability of the genotyped data. Additionally, we will exclude SNPs and samples with a call rate lower than 90% to enhance data quality. After the initial QC, imputation will be performed to infer missing genotypes based on the Haplotype Reference Consortium (HRC) reference panel, using the Helmholtz Munich Imputation Server [37]. Following this, a post-imputation QC process will be conducted to remove variants with poor imputation quality scores or low MAF, as these are more likely to have higher error rates and may provide limited value for the analysis. Additionally, any potential relatedness will be excluded to prevent confounding effects arising from familial relationships.

#### Biobanking.

Whole blood samples will be stored at the FPG Biobank. These samples may be used in future research studies, provided that specific informed consent is obtained.

### Data collection and management

Pseudo-anonimysed data for the study will be collected and managed using REDCap, a secure, web-based application designed for research data capture [38] https://redcap-irccs.policlinicogemelli.it/redcap/. REDCap provides an intuitive interface for validated data entry, tracks data manipulation, and facilitates automated data export for statistical analysis. Access to the platform will be limited to officially registered study personnel, ensuring the integrity and consistency of the data. All datawill be managed in compliance with General Data Protection Regulation (GDPR).

### Statistical methods

#### Sample size estimation.

The study is offered to the general population who meet the previously outlined inclusion criteria. It is estimated that if the intervention will be proposed to approximately 650 citizens, the 70% will agree to participate (N = 455), and 80% of those will complete the study (N = 364). This sample size allows us to detect a mean difference of 3 points (standard deviation = 2) [35] in the LE8 score between the start and the end of the follow-up, with a statistical power greater than 90%.

#### Statistical analysis.

Baseline characteristics of the study population will be summarized using descriptive statistics.

The baseline and final values will be compared to evaluate the effect of the communication of the PRS. The analysis will employ adjusted mixed-effect models for repeated measures to assess significant differences in LE8, LDL-cholesterol and SCORE-2 from baseline to the follow-up time point (T).

We will conduct a comprehensive subgroup analysis to explore potential moderating and mediating effects based on participant characteristics. The subgroup analysis will encompass sociodemographic characteristics (age above or below 55 years, gender, and marital status), ethnicity, socioeconomic status (occupation and related variables), education level, PRS levels (high and not-high), lifestyle category at baseline (favorable, intermediate, or unfavorable), knowledge toward CVDs (present/absent), and psychological status at baseline. The primary objective of this subgroup analysis is to investigate whether the efficacy of the interventions varies across these identified participant subgroups. We aim to understand how sociodemographic factors, genetics, and baseline lifestyle categories may moderate or mediate the effects of the interventions on behavior change and modification of SCORE-2. Statistical analyses will be performed using STATA (StataCorp, USA) and R (https://www.r-project.org/).

### Ethics approval and registration

Ethics approval for this study was obtained from the Ethics Committee of the Fondazione Policlinico Universitario Agostino Gemelli, “Comitato Etico Territoriale Lazio Area 3” (comitatoetico.lazioarea3@policlinicogemelli.it), under approval number: 6732. Any approved amendmentto the protocol or change of plan will be given to each participant via e-mail or telephone. All participants to whom the study will be proposed will be free to accept participation by signing an informed consent form. If they refuse, they will still receive the best possible care. Participants may also freely withdraw from the study at any time without having to provide any justification.

The study has been approved by the Ethical committeeon the 12/12/2024, and has received two amendments on the 10/07/2025 and on the 18/09/2025.

This trial has been registered on ClinicalTrials.gov the 24th of February, 2025 with the identifier: NCT06888466.

## Discussion

This study addresses the growing need for innovative primary prevention strategies, focusing on the potential impact of communicating individualized genetic risk, via PRS, on promoting healthier lifestyles in preventing CVDs.

By integrating genetic predisposition data with conventional risk factors, this work acknowledges that a subset of individuals suffers myocardial infarction despite the absence of notable clinical markers such as hypercholesterolemia, diabetes, or hypertension [39,40]. These cases underscore the importance of exploring non-modifiable factors like polygenic risk, which may better capture a hidden susceptibility in the broader population [41].

Traditional risk prediction models for cardiovascular diseases primarily rely on clinical and lifestyle factors, typically including variables such as age, sex, blood pressure, cholesterol levels, smoking status, and diabetes status. Lifestyle interventions that typically include smoking cessation, balanced nutrition, and regular physical activity, have long been recognized as cornerstone measures for the primary prevention of atherothrombotic events. However, relying on traditional factors can be a limitation, since these models often do not incorporate novel biomarkers, including genetic, social, or environmental factors, which can enhance predictive accuracy [42–44]. Personalizing these strategies to each individual’s genetic profile may enhance risk stratification and intervention success [45,46]. Recently, it has also been suggested that work ability, as assessed through the WAI, along with participants’ anthropometric indices, predicts the risk of CVDs, and verifying this hypothesis could also lead to the identification of additional personalized risk factors [24,47].

Indeed, the European Society of Cardiology has noted that while PRS are not yet recommended for routine clinical practice, they are increasingly being used to inform personalized healthcare decisions [47].

With genomic technology becoming more accessible, PRS-based strategies have shown promising results in identifying those at higher CVD risk, particularly when added to established clinical models; however, evidence remains inconsistent regarding whether or how communicating these results induces sustained lifestyle modifications [47,48].

Some trials suggest that genetic risk disclosure can motivate patients to adopt healthier behaviors, possibly by enhancing perceived personal susceptibility, whereas others report modest or no effect, highlighting the significance of how risk is communicated—counselling approaches, level of detail, and support mechanisms can profoundly shape the patient’s comprehension, emotional response, and subsequent behavior [39,41,49–51].

Indeed, risk communication should be supported by clear, evidence-based strategies to facilitate understanding of PRS and its role in disease prevention, while minimizing misunderstandings or unnecessary anxiety in patients [52,53].

Generating robust clinical evidence on the use of PRS in chronic disease prevention, and specifically in CVD, is therefore paramount to guiding how this tool can be effectively integrated into everyday practice [54,55]. Furthermore, the impact of PRS testing and communication on the workflow of healthcare professionals remains understudied, potentially highlighting areas for improvement for their implementation in clinical practice [56].

At the same time, appropriate risk communication strategies—especially those accounting for differing levels of health literacy—are essential to mitigate the possibility that genetic information could trigger worry or complacency, instead encouraging positive lifestyle adjustments [57]. This becomes feasible by providing healthcare professionals with effective training on conveying genetic risk to healthy individuals, who may be less aware of the importance of preventive [55,58].

In that respect, this study protocol focuses on how best to present PRS results, explore patient acceptance, and measure actual lifestyle change, thus providing insights that could inform larger, randomized studies aimed at refining guidelines for PRS-based interventions in preventive cardiology.

Nevertheless, several methodological limitations should be considered. First, due to the absence of a comparator group in this single arm design it could be challenging to isolate the specific impact of genetic risk disclosure from other confounding factors. Second, the study population consists of relatively healthy population, which may limit the generalizability of findings to more diverse or high-risk populations. Third, the study will rely mostly on self-reported measures for assessing lifestyle changes introduce the possibility of recall or social desirability bias. However, we will also objectively measure LDL-cholesterol levels before and after the intervention, which will help strengthen the study design. Fourth, the follow-up period may not be sufficient to determine whether any observed positive behavioral modifications persist in the long term. Finally, genomic risk stratification itself remains in a dynamic stage of refinement, and PRS may have variable performance across different ancestral groups, highlighting the need for further research on diverse populations and for standardized methods in polygenic risk calculation. Despite these limitations, the study offers a valuable pilot framework for examining whether—when thoughtfully communicated—genetic risk information can motivate individuals to adopt and maintain healthier lifestyles, a finding that could profoundly influence both clinical practice and public health strategies for CVD prevention.

## Supporting information

S1 File 5. SPIRIT_Fillable-checklist-_PHC. (PDF)

S2 File Ethics commettee protocols and approvals. (ZIP)

S3 File Supplementary1_Informed consent_ita and eng. (PDF)

S4 File Supplementary2_questionario. (PDF)
