# Supplementary material for: Polygenic Risk Scores disclosure for cardiovascular prevention: Protocol of the Personalized HeartCare (PHC) trial
Source: PLoS One. 2026 Apr 6;21(4):e0345294. doi: 10.1371/journal.pone.0345294 (PMC13052841; doi:10.1371/journal.pone.0345294)
Supplement: S2 File — (ZIP) [file pone.0345294.s002.zip › Ethics commettee protocols and approvals/Ethics_Committee_Approval_PHC_English_1.pdf]

TERRITORIAL ETHICS COMMITTEE LAZIO AREA 3

(Established by Regional Determination No. G01659 of 10/02/2023)

CET Lazio Area 3

Technical-Scientific Secretariat

Fondazione Policlinico Universitario Agostino Gemelli IRCCS

Università Cattolica del Sacro Cuore

Largo Francesco Vito, 1, 00168 Rome, Italy

comitatoetico.lazioarea3@policlinicogemelli.it

Tel. +39 06/30156124 - 5556

Tax Code and VAT No. 13109681000

REGIONAL HEALTH SERVICE
ID 6732 – NON-PROFIT FUNDED STUDY

To:

Prof. Giovanni Scambia

Director, Department of Life Sciences and Public Health

Prof. Stefania Boccia

Department of Life Sciences and Public Health – Section of Hygiene

Grant Office

Fondazione Policlinico Universitario A. Gemelli IRCCS

Università Cattolica del Sacro Cuore

Meeting of 12 December 2024

Members Present

- Prof. Andrea Bacigalupo, Clinician – Chair
- Dr. Salvatore Accordino, Hospital Pharmacist
- Prof. Massimo Ciccozzi, Biostatistician
- Dr. Antonello Cocchieri, Representative of the healthcare professions involved in the trial
- Prof. Roberto Coppola, Clinician

- Prof. Sebastiano Filetti, Clinician
- Prof. Rosario Francesco Grasso, Clinician – Expert in new technical, diagnostic and therapeutic procedures, invasive and semi-invasive
- Prof. Fiorella Gurrieri, Genetics Expert
- Avv. Filippo Elvino Leone, Legal Expert
- Dr. Giuseppina Loffredi, Representative of patient or citizen associations engaged in health-related issues
- Ing. Francesco Macchia, Clinical Engineer
- Prof. Fabio Midulla, Pediatrician
- Prof. Maria Rita Migliorino, Clinician
- Prof. Maurizio Muscaritoli, Expert in Human Nutrition
- Prof. Pierluigi Navarra, Pharmacologist
- Prof. Claudio Pisanelli, Expert in Medical Devices
- Prof. Saverio Potenza, Forensic Physician
- Prof. Antonio Gioacchino Spagnolo, Bioethics Expert
- Prof. Fabio Valente, General and Community Practitioner

Members Absent

- Avv. Danilo Gallitelli, Insurance Law Expert

All members preliminarily declared that they would abstain from deliberating on any study in which a direct or indirect conflict of interest might exist.

The Territorial Ethics Committee (CET) met on 12 December 2024 to issue its reasoned ethical opinion regarding Study Protocol PHC, promoted by the Università Cattolica del Sacro Cuore and submitted by Prof. Stefania Boccia, concerning the research project:

“Personalised HeartCare (PHC): Innovative approaches for personalised primary prevention of cardiovascular diseases.”

Documentation Reviewed

- Full study protocol (Version 1.0 dated 28/10/2024 – updated following pre-meeting requests)
- Protocol signature page (Version dated 28/10/2024)
- Protocol synopsis (Version 1.0 dated 28/10/2024)
- PI GCP training certificate
- Researcher’s CV (Version dated 24/05/2024)
- Emergency card (Version 1.0 dated 28/10/2024)
- PHC questionnaire
- Application for opinion/Letter of intent (dated 28/10/2024)
- PNC_DARE_Call_Annex 1 – FINAL
- Informed Consent Form (Version 1.0 dated 28/10/2024 – updated following pre-meeting requests)

- Personal Data Processing Consent (Version 1.0 dated 28/10/2024)
- Letter to the General Practitioner (Version 1.0 dated 28/10/2024)
- Investigator's Conflict of Interest Declaration (dated 13/09/2024)
- Documentation for Non-Profit Sponsor Studies

Having Ascertained That

- The protocol is justified with regard to the risk/benefit ratio;
- The protocol is scientifically and ethically justified in terms of rationale and objectives;
- The protocol is justified in terms of study design;
- The protocol is justified regarding the study population;
- The protocol is justified with respect to the information provided to participants and the procedures for obtaining informed consent;
- The protocol is justified with respect to the planned assessment procedures;
- The protocol is justified with respect to the qualifications of the investigator and/or the adequacy of the available facilities and equipment;
- The protocol is justified with respect to additional financial costs for the Institution;
- The protocol is justified with respect to insurance coverage;
- The protocol is justified with respect to sample size and statistical analysis;
- The protocol refers to the current revision of the Declaration of Helsinki and Good Clinical Practice (ICH-GCP) guidelines, Ministerial Decrees of 18 and 19 March 1998 and subsequent amendments, and complies with current regulations.

Favorable Opinion

The Committee issues a FAVORABLE OPINION.

It is recommended that the centre-specific documentation be resubmitted with correction of the co-funding body (MUR rather than the Ministry of Health).

This opinion was expressed unanimously.

The Committee requests to be informed of the start of the study, its progress through an annual report, its completion or possible early termination, and any subsequent protocol amendments.

It is hereby declared that the CET, reconstituted pursuant to Ministerial Decree of 26 January 2023, Ministerial Decree of 30 January 2023, and Regional Determination No. G01659 of 10 February 2023, is organised and operates in compliance with Good Clinical Practice (ICH-GCP) and current legislation.

The study may begin only after issuance of the formal authorization resolution by the affiliated Institution.

Chair of CET Lazio Area 3

Prof. Andrea Bacigalupo
