# Supplementary material for: Polygenic Risk Scores disclosure for cardiovascular prevention: Protocol of the Personalized HeartCare (PHC) trial
Source: PLoS One. 2026 Apr 6;21(4):e0345294. doi: 10.1371/journal.pone.0345294 (PMC13052841; doi:10.1371/journal.pone.0345294)
Supplement: S2 File — (ZIP) [file pone.0345294.s002.zip › Ethics commettee protocols and approvals/Ethics_Committee_Substantial_Amendment_2_PHC_English_3.pdf]

TERRITORIAL ETHICS COMMITTEE LAZIO AREA 3

(Established by Regional Determination No. G01659 of 10/02/2023, No. G07870 of 06/06/2023 and No. G01589 of 10/02/2025)

CET Lazio Area 3

Technical-Scientific Secretariat

Fondazione Policlinico Universitario Agostino Gemelli IRCCS

Università Cattolica del Sacro Cuore

Largo Francesco Vito, 1, 00168 Rome, Italy

comitatoetico.lazioarea3@policlinicogemelli.it

Tel. +39 06/30156124 - 5556

Tax Code and VAT No. 13109681000

REGIONAL HEALTH SERVICE
ID 6732

To:

Prof. Stefania Boccia

Department of Life Sciences and Public Health and

Department of Cardiovascular and Pulmonary Sciences

Fondazione Policlinico Universitario Agostino Gemelli IRCCS, Rome

Università Cattolica del Sacro Cuore, Rome

Meeting of 18 September 2025

Members Present

- Prof. Salvatore Accordino – Hospital Pharmacist
- Prof. Andrea Bacigalupo – Clinician – Chair
- Prof. Massimo Ciccozzi – Biostatistician
- Dr. Antonello Cocchieri – Representative of the healthcare professions involved in the trial
- Prof. Roberto Coppola – Clinician
- Avv. Danilo Gallitelli – Insurance Law Expert
- Prof. Claudio Gasperini – Neurologist

- Prof. Rosario Francesco Grasso – Clinician – Expert in new technical, diagnostic and therapeutic invasive and semi-invasive procedures
- Prof. Fiorella Gurrieri – Genetics Expert
- Avv. Filippo Elvino Leone – Legal Expert
- Dr. Giuseppina Loffredi – Representative of patient or citizen associations engaged in health-related issues
- Ing. Francesco Paolo Macchia – Clinical Engineer
- Prof. Fabio Midulla – Pediatrician
- Prof. Maria Rita Migliorino – Clinician
- Prof. Maurizio Muscaritoli – Expert in Human Nutrition
- Prof. Pierluigi Navarra – Pharmacologist
- Prof. Claudio Pisanelli – Expert in Medical Devices
- Prof. Saverio Potenza – Forensic Physician
- Prof. Antonio Gioacchino Spagnolo – Bioethics Expert
- Prof. Domenico Tarantino – Pharmacist
- Prof. Fabio Valente – General and Community Practitioner

Members Absent

- Prof. Sebastiano Filetti – Clinician

All members preliminarily declared that they would abstain from deliberating on any study in which a direct or indirect conflict of interest might exist.

The Territorial Ethics Committee (CET) met on 18 September 2025 to issue its reasoned ethical opinion on Substantial Amendment No. 2 (Protocol Version 3.0) relating to the research project entitled:

“Personalised HeartCare (PHC): Innovative approaches for personalised primary prevention of cardiovascular diseases.”

Documentation Reviewed

- Cover letter dated 22/07/2025
- Protocol Version 3.0 dated 22/07/2025
- Informed Consent Form for competent subjects Version 3.0 dated 22/07/2025
- PHC Questionnaire Version 3.0 dated 22/07/2025
- PHC Elevated Report Version 1.0 dated 22/07/2025
- PHC Non-Elevated Report Version 1.0 dated 22/07/2025

Favorable Opinion

The Committee issues a FAVORABLE OPINION.

This opinion was expressed unanimously.

It is hereby declared that the CET, reconstituted pursuant to Ministerial Decree of 26 January 2023, Ministerial Decree of 30 January 2023, and Regional Determination No. G01659 of 10 February 2023, is organised and operates in compliance with Good Clinical Practice (ICH-GCP) and current applicable regulations.

Chair of CET Lazio Area 3

Prof. Andrea Bacigalupo
