# Supplementary material for: Polygenic Risk Scores disclosure for cardiovascular prevention: Protocol of the Personalized HeartCare (PHC) trial
Source: PLoS One. 2026 Apr 6;21(4):e0345294. doi: 10.1371/journal.pone.0345294 (PMC13052841; doi:10.1371/journal.pone.0345294)
Supplement: S2 File — (ZIP) [file pone.0345294.s002.zip › Ethics commettee protocols and approvals/PARERE Approval ID 6732_signed.pdf]

REGIONE LAZIO

SISTEMA SANITARIO REGIONALE

COMITATO ETICO TERRITORIALE LAZIO AREA 3

(istituita con determinazione regionale n. G01659 del 10/02/2023)

ID 6732 STUDIO NO PROFIT FINANZIATO

Chiar.mo Prof. Giovanni SCAMBIA
Direttore DIPARTIMENTO DI SCIENZE DELLA VITA E SANITA' PUBBLICA

Gent.ma Prof.ssa Stefania BOCCIA
DIPARTIMENTO DI SCIENZE DELLA VITA E SANITA' PUBBLICA – SEZIONE DI IGIENE

Spett.le Grant Office

Fondazione Policlinico Universitario A. Gemelli IRCCS

UNIVERSITA' CATTOLICA DEL SACRO CUORE

Riunione del 12 Dicembre 2024

Membri Presenti:

Prof. Andrea BACIGALUPO, *Clinico. Presidente*
Dr. Salvatore ACCORDINO, *Farmacista ospedaliero*
Prof. Massimo CICCOZZI, *Biostatistico*
Dr. Antonello COCCHIERI, *Rappresentante dell'area delle professioni sanitarie interessate alla sperimentazione*
Prof. Roberto COPPOLA, *Clinico*
Prof. Sebastiano FILETTI, *Clinico*
Prof. Rosario Francesco GRASSO, *Clinico. Esperto nuove procedure tecniche, diagnostiche e terapeutiche, invasive e semi invasive*
Prof.ssa Fiorella GURRIERI, *Esperto in genetica*

COMITATO ETICO TERRITORIALE LAZIO AREA 3

(istituita con determinazione regionale n. G01659 del 10/02/2023)

Avv. Filippo Elvino LEONE, *Esperto in materia giuridica*
Dott.ssa Giuseppina LOFFREDI, *Rappresentante delle associazioni pazienti o cittadini impegnati sui temi della salute*
Ing. Francesco MACCHIA, *Ingegnere clinico*
Prof. Fabio MIDULLA, *Pediatra*
Prof.ssa Maria Rita MIGLIORINO, *Clinico*
Prof. Maurizio MUSCARITOLI, *Esperto in nutrizione sull'uomo*
Prof. Pierluigi NAVARRA, *Farmacologo*
Prof. Claudio PISANELLI, *Esperto in dispositivi medici*
Prof. Saverio POTENZA, *Medico legale*
Prof. Antonio Gioacchino SPAGNOLO, *Esperto di Bioetica*
Prof. Fabio VALENTE, *Medico di medicina generale e territoriale*

Membri Assenti:

Avv. Danilo GALLITELLI, *Esperto in materia assicurativa*

I componenti hanno preliminarmente dichiarato di non pronunciarsi per quelle sperimentazioni per le quali possa sussistere un conflitto di interessi di tipo diretto o indiretto.

Il Comitato Etico Territoriale (CET), si è riunito il 12 Dicembre 2024 per esprimere il proprio parere etico motivato sullo **studio Prot. PHC promosso dall'Università Cattolica del Sacro Cuore** presentato dalla Prof.ssa Stefania Boccia, relativo alla ricerca **"Personalised HeartCare (PHC): approcci innovativi per la prevenzione primaria personalizzata delle malattie cardiovascolari",**

ESAMINATA

la seguente documentazione:

CET Lazio Area 3
Segreteria Tecnico-Scientifica
Fondazione Policlinico Universitario Agostino Gemelli IRCCS
Università Cattolica del Sacro Cuore
Largo Francesco Vito, 1, 00168 Roma
comitatoetico.lazioarea3@policlinicogemelli.it
T +39 06/30156124 - 5556
C.F e P. IVA 13109681000

- **Protocollo di studio in esteso** (versione *1.0* del *28/10/2024 – aggiornata dopo richieste pre-seduta*)
- **Pagina firma protocollo** (versione del *28/10/2024*)
- **Sinossi del protocollo** (versione *1.0* del *28/10/2024*)
- **Certificato corso GCP del PI**
- **Curriculum del ricercatore** (versione del *24/05/2024*)
- **Emergency card** (versione *1.0* del *28/10/2024*)
- **Questionario PHC**
- **Domanda di parere/lettera di intenti** (datata *28/10/2024*)
- **Avviso PNC_DARE_Allegato 1 - FINAL**
- **Modulo per il consenso informato** (versione *1.0* del *28/10/2024 – aggiornata dopo richieste pre-seduta*)
- **Consenso per il trattamento dei dati personali** (versione *1.0* del *28/10/2024*)
- **Lettera al medico curante** (versione *1.0* del *28/10/2024*)
- **Dichiarazione conflitto di interessi dello sperimentatore** (datata *13/09/2024*)
- **Modulistica Studi con Promotore No Profit**

CONSTATATO CHE

il protocollo presentato:

- è giustificato quanto al rapporto rischi/benefici;
- è giustificato scientificamente ed eticamente quanto al razionale e agli obiettivi;
- è giustificato quanto al disegno sperimentale;
- è giustificato quanto ai soggetti di sperimentazione;
- è giustificato quanto alle informazioni fornite ai soggetti e alle modalità di richiesta del consenso;
- è giustificato quanto agli esami valutativi previsti;
- è giustificato quanto alla qualificazione del ricercatore e/o delle strutture e attrezzature disponibili;

- è giustificato quanto ai costi economici aggiuntivi per l'Ente;
- è giustificato quanto alle garanzie assicurative;
- è giustificato quanto alla numerosità campionaria e all'indagine statistica;
- fa riferimento ai codici deontologici (in particolare alla revisione corrente della dichiarazione di Helsinki e/o alle Norme di Buona Pratica Clinica (ICH-GCP) secondo l'all. 1 al D.M. 15.7.97) ed ai D.M. 18 e 19 marzo '98 e successive modificazioni ed integrazioni ed è compliante con la normativa vigente;

ESPRIME PARERE FAVOREVOLE

Si raccomanda di ripresentare la modulistica centro-specifica corretta nell'indicazione dell'Ente cofinanziatore (MUR piuttosto che Ministero della Salute).

Il presente parere è stato espresso all'unanimità.

Si richiede che questo CET venga informato dell'inizio della sperimentazione, del suo svolgimento con una relazione annuale e della sua conclusione o eventuale interruzione. Inoltre dovrà essere informato di ogni successivo emendamento al protocollo.

Si dichiara che il CET, ricostituito ai sensi del DM 26 Gennaio 2023, del DM 30 Gennaio 2023, della Determinazione Regionale n. G01659 del 10 Febbraio 2023 è organizzato ed opera nel rispetto delle norme di buona pratica clinica (GCP-ICH) e degli adempimenti previsti dalla normava vigente.

Si fa presente che lo studio potrà avere inizio solo a seguito del rilascio della delibera autorizzativa da parte dell'Ente di appartenenza.

Il Presidente del CET Lazio Area 3
Prof. Andrea Bacigalupo

ANDREA BACIGALUPO
17.12.2024 15:55:45
GMT+01:00

CET Lazio Area 3

Segreteria Tecnico-Scientifica
Fondazione Policlinico Universitario Agostino Gemelli IRCCS
Università Cattolica del Sacro Cuore
Largo Francesco Vito, 1, 00168 Roma
comitatoetico.lazioarea3@policlinicogemelli.it
T +39 06/30156124 - 5556
C.F e P. IVA 13109681000
