# Supplementary material for: Polygenic Risk Scores disclosure for cardiovascular prevention: Protocol of the Personalized HeartCare (PHC) trial
Source: PLoS One. 2026 Apr 6;21(4):e0345294. doi: 10.1371/journal.pone.0345294 (PMC13052841; doi:10.1371/journal.pone.0345294)
Supplement: S2 File — (ZIP) [file pone.0345294.s002.zip › Ethics commettee protocols and approvals/PARERE EM 1 ID 6732_signed.pdf]

REGIONE LAZIO

SISTEMA SANITARIO REGIONALE

COMITATO ETICO TERRITORIALE LAZIO AREA 3

(istituito con determinazione regionale n. G01659 del 10/02/2023, n. G07870 del 6/06/2023 e n. G01589 del 10/02/2025)

ID 6732

Gent.ma Prof.ssa Stefania BOCCIA
Dipartimento di Scienze della Vita e Sanità Pubblica ed
il Dipartimento di Scienze cardiovascolari e pneumologiche
Fondazione Policlinico Universitario Agostino Gemelli IRCCS Roma
Università Cattolica del Sacro Cuore Roma

Riunione del 10 LUGLIO 2025

Membri presenti:
Prof. Salvatore Accordino_Farmacista ospedaliero
Prof. Andrea Bacigalupo_Clinico_Presidente
Prof. Roberto Coppola_Clinico
Prof. Sebastiano Filetti_Clinico
Avv. Danilo Gallitelli_Esperto in materia assicurativa
Claudio Gasperini Neurologo
Prof. Rosario Francesco Grasso _Clinico. Esperto nuove procedure tecniche, diagnostiche e terapeutiche invasive e semi invasive
Prof.ssa Fiorella Gurrieri_Esperto in genetica
Avv.Filippo Elvino Leone_Esperto in materia giuridica
Dott.ssa Giuseppina Loffredi_Rappr.delle associazioni pazienti o cittadini impegnati sui temi della salute
Prof. Fabio Midulla_Pediatra
Prof.ssa Maria Rita Migliorino_Clinico
Prof. Maurizio Muscaritoli_Esperto in nutrizione sull’uomo
Prof. Pierluigi Navarra_Farmacologo
Prof. Claudio Pisanelli_Esperto in dispositivi medici
Prof. Saverio Potenza_Medico legale
Prof. Antonio Gioacchino Spagnolo_Esperto di bioetica
Domenico Tarantino Farmacista
Prof. Fabio Valente_Medico di medicina generale e territoriale

CET Lazio Area 3

Segreteria Tecnico-Scientifica
Fondazione Policlinico Universitario Agostino Gemelli IRCCS
Università Cattolica del Sacro Cuore
Largo Francesco Vito, 1, 00168 Roma
comitatoetico.lazioarea3@policlinicogemelli.it
T +39 06/30156124 - 5556
C.F e P. IVA 13109681000

COMITATO ETICO TERRITORIALE LAZIO AREA 3

(istituito con determinazione regionale n. G01659 del 10/02/2023, n. G07870 del 6/06/2023 e n. G01589 del 10/02/2025)

Membri assenti:
Prof. Massimo Ciccozzi_Biostatistico
Dott. Antonello Cocchieri_Rappr.dell'area delle professioni sanitarie interessate alla sperimentazione
Ing.Francesco Paolo Macchia_Ingegnere clinico

I componenti hanno preliminarmente dichiarato di non pronunciarsi per quelle sperimentazioni per le quali possa sussistere un conflitto di interessi di tipo diretto o indiretto.

Il Comitato Etico Territoriale (CET), si è riunito il 10 LUGLIO 2025 per esprimere il proprio parere etico motivato sull'**Emendamento Sostanziale 1 del 24/06/2025** relativo alla ricerca dal titolo "*Personalised HeartCare (***PHC***): approcci innovativi per la prevenzione primaria personalizzata delle malattie cardiovascolari",*

ESAMINATA

la seguente documentazione:

- Lettera di trasmissione del 24/06/2025
- Protocollo PHC Versione V2 del 24.06.2025
- Sinossi Versione V2 del 24.06.2025
- Consenso informato per soggetti capaci Versione V2 del 24.06.2025
- Modulistica centro specifica Versione V2 del 24.06.2025
- Questionario PHC Versione V2 del 24.06.2025
- Locandina PHC Versione V2 del 24.06.2025

ESPRIME PARERE FAVOREVOLE CONDIZIONATO

con le seguenti prescrizioni: *come indicato nel Protocollo a pagina 12 "*È prevista la raccolta di campioni ematici (sangue intero), che verranno riposti presso la Biobanca di FPG – Biobanca. Tali campioni potranno essere utilizzati per eventuali, ulteriori studi futuri previo consenso specifico*", si chiede di allegare tale consenso.*

CET Lazio Area 3

Segreteria Tecnico-Scientifica
Fondazione Policlinico Universitario Agostino Gemelli IRCCS
Università Cattolica del Sacro Cuore
Largo Francesco Vito, 1, 00168 Roma
comitatoetico.lazioarea3@policlinicogemelli.it
T +39 06/30156124 - 5556
C.F e P. IVA 13109681000

COMITATO ETICO TERRITORIALE LAZIO AREA 3

(istituito con determinazione regionale n. G01659 del 10/02/2023, n. G07870 del 6/06/2023 e n. G01589 del 10/02/2025)

Il presente parere è stato espresso all'unanimità.

È necessario far pervenire entro 60 giorni dalla ricezione del presente parere, pena il decadimento dell'intera istruttoria, la documentazione modificata secondo quanto richiesto, prima dell'inizio effettivo dello studio, per avere una presa d'atto finale, per la quale non sarà necessario attendere la seduta plenaria.

Si dichiara che il CET, ricostituito ai sensi del DM 26 Gennaio 2023, del DM 30 Gennaio 2023, della Determinazione Regionale n. G01659 del 10 Febbraio 2023 è organizzato ed opera nel rispetto delle norme di buona pratica clinica (GCP-ICH) e degli adempimenti previsti dalla normava vigente.

Il Presidente del CET Lazio Area 3
Prof. Andrea Bacigalupo

ANDREA
BACIGALUPO
16.07.2025
10:31:33
GMT+02:00

CET Lazio Area 3

Segreteria Tecnico-Scientifica
Fondazione Policlinico Universitario Agostino Gemelli IRCCS
Università Cattolica del Sacro Cuore
Largo Francesco Vito, 1, 00168 Roma
comitatoetico.lazioarea3@policlinicogemelli.it
T +39 06/30156124 - 5556
C.F e P. IVA 13109681000
