# Supplementary material for: Polygenic Risk Scores disclosure for cardiovascular prevention: Protocol of the Personalized HeartCare (PHC) trial
Source: PLoS One. 2026 Apr 6;21(4):e0345294. doi: 10.1371/journal.pone.0345294 (PMC13052841; doi:10.1371/journal.pone.0345294)
Supplement: S2 File — (ZIP) [file pone.0345294.s002.zip › Ethics commettee protocols and approvals/PARERE EM 2 ID 6732_signed.pdf]

REGIONE LAZIO

SISTEMA SANITARIO REGIONALE

COMITATO ETICO TERRITORIALE LAZIO AREA 3

(istituito con determinazione regionale n. G01659 del 10/02/2023, n. G07870 del 6/06/2023 e n. G01589 del 10/02/2025)

ID 6732

Gent.ma Prof.ssa Stefania BOCCIA
Dipartimento di Scienze della Vita e Sanità Pubblica ed
il Dipartimento di Scienze cardiovascolari e pneumologiche
Fondazione Policlinico Universitario Agostino Gemelli IRCCS Roma
Università Cattolica del Sacro Cuore Roma

Riunione del 18 SETTEMBRE 2025

Membri presenti:
Prof. Salvatore Accordino_Farmacista ospedaliero
Prof. Andrea Bacigalupo_Clinico_Presidente
Prof. Massimo Ciccozzi_Biostatistico
Dott. Antonello Cocchieri_Rappr.dell'area delle professioni sanitarie interessate alla sperimentazione
Prof. Roberto Coppola_Clinico
Avv. Danilo Gallitelli_Esperto in materia assicurativa
Prof. Claudio Gasperini Neurologo
Prof. Rosario Francesco Grasso _Clinico. Esperto nuove procedure tecniche, diagnostiche e terapeutiche invasive e semi invasive
Prof.ssa Fiorella Gurrieri_Esperto in genetica
Avv.Filippo Elvino Leone_Esperto in materia giuridica
Dott.ssa Giuseppina Loffredi_Rappr.delle associazioni pazienti o cittadini impegnati sui temi della salute
Ing.Francesco Paolo Macchia_Ingegnere clinico
Prof. Fabio Midulla_Pediatra
Prof.ssa Maria Rita Migliorino_Clinico

CET Lazio Area 3
Segreteria Tecnico-Scientifica
Fondazione Policlinico Universitario Agostino Gemelli IRCCS
Università Cattolica del Sacro Cuore
Largo Francesco Vito, 1, 00168 Roma
comitatoetico.lazioarea3@policlinicogemelli.it
T +39 06/30156124 - 5556
C.F e P. IVA 13109681000

COMITATO ETICO TERRITORIALE LAZIO AREA 3

(istituito con determinazione regionale n. G01659 del 10/02/2023, n. G07870 del 6/06/2023 e n. G01589 del 10/02/2025)

Prof. Maurizio Muscaritoli_Esperto in nutrizione sull'uomo
Prof. Pierluigi Navarra_Farmacologo
Prof. Claudio Pisanelli_Esperto in dispositivi medici
Prof. Saverio Potenza_Medico legale
Prof. Antonio Gioacchino Spagnolo_Esperto di bioetica
Prof. Domenico Tarantino Farmacista
Prof. Fabio Valente_Medico di medicina generale e territoriale

Membri assenti:
Prof. Sebastiano Filetti_Clinico

I componenti hanno preliminarmente dichiarato di non pronunciarsi per quelle sperimentazioni per le quali possa sussistere un conflitto di interessi di tipo diretto o indiretto.

Il Comitato Etico Territoriale (CET), si è riunito il 18 SETTEMBRE 2025 per esprimere il proprio parere etico motivato sull'**Emendamento sostanziale 2 Prot 3.0**, relativo alla ricerca dal titolo "*Personalised HeartCare (PHC): approcci innovativi per la prevenzione primaria personalizzata delle malattie cardiovascolari*"

ESAMINATA

la seguente documentazione:

- Lettera di trasmissione del 22/07/2025
- Protocollo v3.0 del 22/07/2025
- Consenso Informto per soggetti capaci v3.0 del 22/07/2025
- Questionario PHC v3.0 del 22/07/2025
- Report PHC elevato v1.0 del 22/07/2025

COMITATO ETICO TERRITORIALE LAZIO AREA 3

(istituito con determinazione regionale n. G01659 del 10/02/2023, n. G07870 del 6/06/2023 e n. G01589 del 10/02/2025)

- Report PHC non elevato v1.0 del 22/07/2025

ESPRIME PARERE FAVOREVOLE

Il presente parere è stato espresso all'unanimità.

Si dichiara che il CET, ricostituito ai sensi del DM 26 gennaio 2023, del DM 30 gennaio 2023, della Determinazione Regionale n. G01659 del 10 febbraio 2023 è organizzato ed opera nel rispetto delle norme di buona pratica clinica (GCP-ICH) e degli adempimenti previsti dalla normava vigente.

Il Presidente del CET Lazio Area 3
Prof. Andrea Bacigalupo

ANDREA
BACIGALUPO
24.09.2025
10:45:20
GMT+02:00

CET Lazio Area 3

Segreteria Tecnico-Scientifica
Fondazione Policlinico Universitario Agostino Gemelli IRCCS
Università Cattolica del Sacro Cuore
Largo Francesco Vito, 1, 00168 Roma
comitatoetico.lazioarea3@policlinicogemelli.it
T +39 06/30156124 - 5556
C.F e P. IVA 13109681000
