# Supplementary material for: Polygenic Risk Scores disclosure for cardiovascular prevention: Protocol of the Personalized HeartCare (PHC) trial
Source: PLoS One. 2026 Apr 6;21(4):e0345294. doi: 10.1371/journal.pone.0345294 (PMC13052841; doi:10.1371/journal.pone.0345294)
Supplement: S2 File — (ZIP) [file pone.0345294.s002.zip › Ethics commettee protocols and approvals/Protocollo_PHC_v.1.0 del 28.10.2024 eng.pdf]

PROTOCOL

Version 1.0 dated 28/01/2024

"Personalized HeartCare (PHC): innovative approaches for personalized primary prevention of cardiovascular disease"

ACRONYM: PHC

Principal Investigator (PI): Prof. Stefania Boccia

Sub-Investigators:

- Dr. Roberta Pastorino (Co-Proponent)
- Prof. Giovanna Liuzzo (Co-Proponent)

Promoter: Catholic University of the Sacred Heart, Largo Francesco Vito 1, 00168, Rome (Italy)

Funding: Non-profit co-funded

UNITS INVOLVED

- **Catholic University of the Sacred Heart**
  - **"Department of Life Sciences and Public Health - Section of General and Applied Hygiene 1 (IG1)":**
    - Prof. Stefania Boccia (Proponent)
    - Dr. Roberta Pastorino (Co-Proponent)
    - Dr. Tina Pasciuto
    - Dr. Luigi Russo
    - Dr. Luca Proto
    - Dr. Sara Farina
    - Dr. Eleonora Pascucci
- **IRCCS A. Gemelli University Hospital Foundation:**
  - **"Department of Cardiovascular and Pulmonary Sciences - Section of Cardiovascular Diseases":**
    - Prof. Liuzzo Giovanna (Co-Proponent)
    - Dr. Anna Severino

PROJECT DESCRIPTION

Background and rationale of the study

Over the last decade, personalized medicine (PM) has undergone considerable development, sparking a veritable revolution in healthcare [1]. By combining genetic information, environmental and behavioral characteristics, and the specific socioeconomic and cultural context of each individual, the aim is to identify the best treatment, tailored to their unique characteristics [2,3].

In this context, Personalized Prevention has been developed, with the aim of identifying the best approaches to prevent the onset and promote early diagnosis of major diseases. A change in strategy, moving away from a system based on treating individuals, is necessary in light of the aging of the general population and the increase in age-related diseases [1,4]. The National Recovery and Resilience Plan (PNRR), approved in 2021 as part of the European Next Generation EU program, has two objectives for the national healthcare system: on the one hand, to modernize the country, especially by promoting digital healthcare and technological development, and on the other, to promote strategies for early diagnosis and disease prevention [5,6].

Cardiovascular disease (CVD) is the leading cause of mortality and morbidity in Europe and is due to a combination of unhealthy lifestyles, environmental factors, and genetic predisposition [7]. There are numerous genetic variants associated with the onset of cardiovascular disease, and their impact can be measured using the Polygenic Risk Score (PRS), which estimates each individual's predisposition to developing the disease by weighting the influence of each individual variant. These scores can be used to implement personalized preventive strategies, tailored to each patient's risk profile, to delay or prevent the onset of disease [8,9,10,11,12].

Over the last two decades, the prevalence of risk factors for cardiovascular disease has increased significantly. Unhealthy diets, physical inactivity, and cigarette smoking are particularly high among young people aged 18-35, predisposing them to the development of cardiovascular disease [13], with dramatic consequences for the future of the national health system. The latest data predict a 20% increase in mortality by 2030 compared to 2019 data, and an increase in DALYs (Disability Adjusted Life Years) [14].

In light of this epidemic of cardiovascular disease, prevention becomes a fundamental objective for global healthcare systems. It is well known that improving one's eating habits, increasing physical activity, and quitting smoking reduce the risk of developing cardiovascular disease and, consequently, lead to a decreased burden in older age groups. [15,16]

The aim of this study is to encourage a healthy lifestyle in healthy individuals with low traditional cardiovascular risk by communicating their genetic predisposition risk profile, measured by calculating their PRS. This study would allow for the evaluation of innovative personalized approaches, with the ultimate goal of reducing the burden of future cardiovascular disease and the associated costs.

OBJECTIVES

General objective

The general objective of the study is to evaluate changes in lifestyle after communicating the Polygenic Risk Score, measured using a validated questionnaire.

Primary objective

- To evaluate the effectiveness of communicating the Polygenic Risk Score in modifying participants' lifestyle.

Secondary objective

- Assess the feasibility of introducing the Polygenic Risk Score into the patient care pathway

ENDPOINTS

Primary endpoint

- Pre-post change in lifestyle score, measured using Life Essentials' 8, at baseline and at final follow-up [17,18,19,20]

Secondary endpoints

- Number of participants who quit or reduced smoking at final follow-up.
- Number of participants who changed their eating pattern at final follow-up.
- Number of participants who quit or reduced alcohol consumption at final follow-up.
- Number of participants who increased their physical activity score
- Acceptability of the Polygenic Risk Score test for patients
- Acceptability of the pathway for both the hospital and patients.
- Compliance with the study, measured by the percentage of subjects who complete all follow-ups
- Compliance with interventions, measured by the number of subjects who adhere to the frequency, content, and duration of the proposed interventions

METHODS

Study design

Single-arm, single-center experimental study in the cohort of Policlinico Gemelli staff enrolled at the Cardiology Department outpatient clinics.

Population

The target population is the staff of the Policlinico Gemelli hospital who were asked to participate in the CVRISK IT study but were not eligible for the study or refused to participate.
Recruitment will be carried out at the cardiovascular prevention clinics, in collaboration with the Cardiology Unit.

Duration of the study

12 months

Inclusion criteria

- Traditional cardiovascular risk, measured with SCORE 2 (Low risk (< 2.5%); Moderate risk (between 2.5% and 5%); High risk (between 5% and 10%) or with SCORE 2-OP (Moderate risk (< 7.5%); High risk (between 7.5% and 15%)
- Availability of blood chemistry tests within the previous 6 months
- Subjects aged ≥ 18 years.

Exclusion criteria

- Very high cardiovascular risk measured with SCORE 2 (very high risk > 10%) or SCORE 2-OP (very high risk > 15%)
- Diabetes
- Familial hypercholesterolemia
- Previous cardiovascular events

Procedures

The study includes an enrollment phase (T0) during the first visit of the CVRISK-IT multicenter clinical trial. This study involves the enrollment of subjects belonging to any cardiovascular risk category measured with SCORE-2 or SCORE 2 OP. However, only low-, intermediate-, and high-risk subjects will enter phase two of the study and will be randomized, while very high-risk subjects will not be randomized but will be placed on a wait list. Subjects who are not at very high risk (SCORE-2 < 10% or SCORE 2 OP <15%) who refuse to enter the study or cannot be enrolled for other reasons will be offered the opportunity to participate in the PHC pilot study.

Subjects who agree to participate in the PHC study will be given the specific informed consent form for this study and will undergo a baseline assessment, including questionnaires to be completed and blood sampling for PRS assessment. The results of the PRS assessment

will be communicated in an informative visit about one month later (T1). Finally, subjects will be re-evaluated six months (T2) after the informative visit to reassess their lifestyle.

Specifically, at the time of enrollment (T0), all participants will sign the informed consent form and undergo a comprehensive assessment:

- They will complete the LE'8 questionnaire, which includes information on socioeconomic status and lifestyle, in particular smoking status, alcohol consumption, eating habits, sleeping patterns, and physical activity.

- undergo a complete medical examination to record biometric data (such as BMI, body circumference), heart rate, and blood pressure.

- Patients will provide blood tests performed independently in the last six months, reporting lipid profile values (total cholesterol, HDL and LDL cholesterol, triglycerides), CRP, and glycemic or glycated hemoglobin, to calculate the SCORE-2 / SCORE 2-OP score.

- Blood samples will be taken for analysis and calculation of the genetic predisposition profile to developing cardiovascular disease, using the Polygenic Risk Score.

A lifestyle score will be calculated for each participant based on the LE8 score.

During the informative online visit (T1), the lifestyle score will be explained. Participants will receive personalized written preventive advice to reduce unhealthy behaviors and modify their lifestyle. The PRS results will also be disclosed to all participants and the risk profile category will be provided, along with all relevant information. At the six-month follow-up from T1 (T2), participants will receive a complete final assessment, as at T0, which includes the submission of the lifestyle questionnaire and the recalculation of the lifestyle category, as well as biometric tests (weight, BMI, body circumference, heart rate, blood pressure). Blood tests will not be repeated.

DATA COLLECTION, RECORDING, AND STATISTICAL ANALYSIS

Questionnaire

The study questionnaire is divided into several sections.

The lifestyle questionnaire (Life's Essential 8) will be administered at T0 and T2, and after completion, will provide a lifestyle score that will classify the patient into one of three categories (favorable, intermediate, unfavorable). The questionnaire has been validated on the European population and has already been used in several clinical studies. Its scale ranges from 0 to 100 [20]. It is divided into several sections, structured as follows:

General lifestyle: Smoking habits, alcohol consumption, physical activity, and amount of sleep
Diet: Types of foods included in one's diet
Health factors: BMI, cholesterol, blood pressure, and blood sugar

Questionnaire on personal and family history, socioeconomic and professional status, and demographic information, administered at T0.

Questionnaire on the acceptability of the intervention to doctors and patients, administered at T2.

Questionnaire on values and preferences regarding the use of new technologies [21], administered at T2.

FACToR questionnaire, a revised version of the MICRA questionnaire, to assess the reaction to genetic testing [22][23], administered at T2.

A questionnaire for assessing anxiety levels (GAD-7) [24], administered at T0 and T2.

Interventions

- Blood samples collected for the calculation of the genetic predisposition profile will be sent to the Hygiene Section. Here, DNA extraction and Subsequent genotyping using the GeneTitan™ MC Fast Scan Instrument from Thermo Fisher Scientific, and PRS calculation using microarray. Based on the results, individuals will be classified into three main categories of CVD risk (high risk, intermediate risk, low risk).

Storage of Biological Material

There are no plans to collect or store biological samples in a biobank.

Data recording

Study data will be collected and managed using REDCap, an electronic data capture tool available at FPG (https://redcap-irccs.policlinicogemelli.it/). REDCap (Research Electronic Data Capture) is a secure, web-based application designed to support data capture for research studies. It provides:

- An intuitive interface for validated data entry;
- Monitoring of data manipulation and export;
- Automated export procedures for seamless downloading of data to common statistical packages**;**
- Procedures for importing data from external sources.

All technical solutions useful for system validation will be implemented with particular attention to data integrity, consistency, and completeness. Only persons officially registered as study investigators or data managers will receive a user login to access the web platform and enter/manage data. Finally, the data will be exported in pseudo-anonymized form for statistical analysis.

CONFIDENTIALITY OF INFORMATION

The personal data of enrolled patients will be processed in accordance with the provisions of Legislative Decree 196/03 and all applicable regulations. Access to samples will be limited to the research manager and his collaborators.

STATISTICAL ANALYSIS

Sample calculation

The study will be proposed to all employees of the Policlinico Gemelli who meet the inclusion criteria listed above. It is estimated that out of a total of approximately 650 employees who will be offered the intervention, 70% will participate in the project (N=455) and of these, 80% will complete the study (N=364). This sample size allows us to detect with a power greater than 90% an average difference of LE8 equal to 3 points (standard deviation=2) between the end and the beginning of the follow-up.

Statistical Analysis

The statistical analysis involves the application of descriptive statistics to describe the sample analyzed in terms of personal data, lifestyles, health factors, blood chemistry parameters collected at baseline, and attitudes. Changes in lifestyle will be analyzed using adjusted mixed-effects models for repeated measures. A potential list of moderators and mediators of the relationship between intervention and behavioral change (such as sociodemographic characteristics, ethnicity, socioeconomic status, education, PRS levels) will be analyzed to evaluate the mechanisms that explain why the intervention may or may not lead to change, i.e., whether people with certain characteristics may benefit more or less from it. Statistical analyses will be conducted using STATA (StataCorp, USA) and R.

REFERENCES

1. Beccia F. et al, An overview of Personalized Medicine landscape and policies in the European Union. European Journal of Public Health, November 1, 2022, 32(6):844-851 https://doi.org/10.1093/eurpub/ckac103 PMID: 36305782 PMCID: PMC9713394
2. PROPHET a PeRsOnalised Prevention roadmap for the future HEalThcare - https://prophetproject.eu/
3. EU Health Policy https://health.ec.europa.eu/eu-health-policy/overview_en
4. Personalized Medicine - European Commission (europa.eu) - Personalized medicine - European Commission (europa.eu)
5. PNRR – Italian Government Presidency of the Council of Ministers - PNRR_0.pdf (governo.it)
6. Next Generation Italia, government plan approved - Next Generation Italia, government plan approved (innovazione.gov.it)
7. About Cardiovascular Disease in ESC Member Countries; (Fact sheets for Press (escardio.org))
8. Knowles, J. W., Zarafshar, S., Pavlovic, A., Goldstein, B. A., Tsai, S., Li, J., McConnell, M. V., Absher, D., Ashley, E. A., Kiernan, M., Ioannidis, J. P. A., & Assimes, T. L. (2017). Impact of a Genetic Risk Score for Coronary Artery Disease on Reducing Cardiovascular Risk: A Pilot Randomized Controlled Study. Frontiers in cardiovascular medicine, 4, 53. https://doi.org/10.3389/fcvm.2017.00053
9. Widén, E., Junna, N., Ruotsalainen, S., Surakka, I., Mars, N., Ripatti, P., Partanen, J. J., Aro, J., Mustonen, P., Tuomi, T., Palotie, A., Salomaa, V., Kaprio, J., Partanen, J., Hotakainen, K., Pöllänen, P., & Ripatti, S. (2022). How Communicating Polygenic and Clinical Risk for Atherosclerotic Cardiovascular Disease Impacts Health Behavior: an Observational Follow-up Study. Circulation. ac and precision medicine, 15(2), e003459. https://doi.org/10.1161/CIRCGEN.121.003459
10. Fenton, G. L., Smit, A. K., Keogh, L., & Cust, A. E. (2019). Exploring the emotional and behavioral reactions to receiving personalized melanoma genomic risk information: a qualitative study. The British journal of dermatology, 180(6), 1390–1396. https://doi.org/10.1111/bjd.17582
11. Lambert, S. A., Abraham, G., & Inouye, M. (2019). Towards clinical utility of polygenic risk scores. *Human molecular genetics*, *28*(R2), R133–R142. https://doi.org/10.1093/hmg/ddz187
12. Lloyd-Jones DM, Allen NB, Anderson CAM, et al. Life's Essential 8: Updating and Enhancing the American Heart Association's Construct of Cardiovascular Health: A Presidential Advisory From the American Heart Association. Circulation. 2022;146(5):e18-e43. doi:10.1161/CIR.0000000000001078
13. Charlotte Andersson 1 2, Ramachandran S Vasan, Epidemiology of cardiovascular disease in young individuals, Nature Reviews Cardiology, 2018 Apr;15(4):230-240. doi: 10.1038/nrcardio.2017.154. Epub 2017 Oct 12. PMID: 29022571, DOI: 10.1038/nrcardio.2017.154 - https://pubmed.ncbi.nlm.nih.gov/29022571/
14. Wang H, Liu J, Feng Y, Ma A, Wang T. The burden of cardiovascular diseases attributable to metabolic risk factors and its change from 1990 to 2019: a systematic analysis and prediction. Front

Epidemiol. 2023 May 25;3:1048515. doi: 10.3389/fepid.2023.1048515. PMID: 38455920; PMCID: PMC10910969.

15. Kiang Liu, Martha L. Daviglus, Catherine M. Loria, Laura A. Colangelo, Bonnie Spring, Arlen C. Moller, and Donald M. Lloyd-Jones, Healthy Lifestyle through Young Adulthood and Presence of Low Cardiovascular Disease Risk Profile in Middle Age: The Coronary Artery Risk Development in (Young) Adults (CARDIA) Study, Circulation. 2012 Feb 28; 125(8): 996–1004. doi: 10.1161/CIRCULATIONAHA.111.060681, PMID: 22291127, PMCID: PMC3353808
16. Charlotte Andersson & Ramachandran S. Vasan, Epidemiology of cardiovascular disease in young individuals, Nat Rev Cardiol 15, 230–240 (2018). https://doi.org/10.1038/nrcardio.2017.154
17. Petermann-Rocha F, Deo S, Celis-Morales C, et al. An Opportunity for Prevention: Associations Between the Life's Essential 8 Score and Cardiovascular Incidence Using Prospective Data from UK Biobank. *Curr Probl Cardiol*. 2023;48(4):101540. doi:10.1016/j.cpcardiol.2022.101540
18. He P, Zhang Y, Ye Z, et al. A healthy lifestyle, Life's Essential 8 scores and new-onset severe NAFLD: A prospective analysis in UK Biobank. *Metabolism*. 2023;146:155643. doi:10.1016/j.metabol.2023.155643
19. Zhang J, Chen G, Habudele Z, et al. Relation of Life's Essential 8 to the genetic predisposition for cardiovascular outcomes and all-cause mortality: results from a national prospective cohort. *Eur J Prev Cardiol*. 2023;30(15):1676-1685. doi:10.1093/eurjpc/zwad179
20. Isiozor NM, Kunutsor SK, Voutilainen A, Laukkanen JA. Life's Essential 8 and the risk of cardiovascular disease death and all-cause mortality in Finnish men. *Eur J Prev Cardiol*. 2023;30(8):658-667. doi:10.1093/eurjpc/zwad040
21. Rosenstock IM. The Health Belief Model and Preventive Health Behavior. Health Education Monographs. 1974;2(4):354-386. doi:10.1177/109019817400200405
22. Cella, David et al. "A brief assessment of concerns associated with genetic testing for cancer: the Multidimensional Impact of Cancer Risk Assessment (MICRA) questionnaire." *Health psychology : official journal of the Division of Health Psychology, American Psychological Association* vol. 21,6 (2002): 564-72.
23. Li, Meng et al. "The Feelings About genomiC Testing Results (FACToR) Questionnaire: Development and Preliminary Validation." *Journal of genetic* counseling vol. 28,2 (2019): 477-490. doi:10.1007/s10897-018-0286-9
24. Spitzer RL, Kroenke K, Williams JBW, Löwe B. A Brief Measure for Assessing Generalized Anxiety Disorder: The GAD-7. Arch Intern Med. 2006;166(10):1092–1097. doi:10.1001/archinte.166.10.1092)
