# Supplementary material for: Polygenic Risk Scores disclosure for cardiovascular prevention: Protocol of the Personalized HeartCare (PHC) trial
Source: PLoS One. 2026 Apr 6;21(4):e0345294. doi: 10.1371/journal.pone.0345294 (PMC13052841; doi:10.1371/journal.pone.0345294)
Supplement: S2 File — (ZIP) [file pone.0345294.s002.zip › Ethics commettee protocols and approvals/Protocollo_PHC_v.2.0 del 24.06.2025.pdf]

PROTOCOLLO

Versione 2.0 del 24/06/2025

"Personalised HeartCare (PHC): approcci innovativi per la prevenzione primaria personalizzata delle malattie cardiovascolari"

ACRONIMO: PHC

Principal Investigator (PI): Prof.ssa Stefania Boccia

Sub-Investigators:

- Dott.ssa Roberta Pastorino (Co-Proponente)
- Prof.ssa Giovanna Liuzzo (Co-Proponente)

Promotore: Università Cattolica del Sacro Cuore, Largo Francesco Vito 1, 00168, Rome (Italy)

Funding: No Profit co-finanziato

UNITA' COINVOLTE

- **Università Cattolica del Sacro Cuore**
    - **"Dipartimento di Scienze della Vita e Sanità Pubblica - Sezione di Igiene Generale e Applicata 1 (IG1)":**
        - Prof.ssa Stefania Boccia (Proponente)
        - Dott.ssa Roberta Pastorino (Co-Proponente)
        - Dott.ssa Tina Pasciuto
        - Dott. Luigi Russo
        - Dott. Luca Proto
        - Dott.ssa Sara Farina
        - Dott.ssa Eleonora Pascucci
- **IRCCS Fondazione Policlinico Universitario A. Gemelli:**
    - **"Dipartimento di Scienze cardiovascolari e pneumologiche - Sezione di Malattie dell'apparato cardiovascolare":**
        - Prof.ssa Liuzzo Giovanna (Co-Proponente)
        - Dott.ssa Anna Severino

- **UOC – Chimica, Biochimica e Biologia Molecolare Clinica:**

    - **"Dipartimento di Scienze di laboratorio ed ematologiche"**

    - Prof. Andrea Urbani

- **"Dipartimento di Sicurezza e Bioetica"**
    - Prof. Ivo Iavicoli

DESCRIZIONE DEL PROGETTO

Background e razionale dello studio

Nell'ultimo decennio la medicina personalizzata (Personalized Medicine, PM) ha avuto un notevole sviluppo, avviando una vera e propria rivoluzione nell'ambito sanitario [1]. Attraverso la combinazione di informazioni genetiche, delle caratteristiche ambientali e comportamentali, del contesto socioeconomico e culturale specifico di ciascun individuo, si pone l'obiettivo di individuare il miglior trattamento, fatto su misura sulle sue caratteristiche uniche [2,3].

In questo contesto, si è sviluppata la Prevenzione Personalizzata, il cui obiettivo è individuare i migliori approcci per prevenire l'insorgenza e favorire la diagnosi precoce delle principali malattie. Un cambiamento di strategia, passando da un sistema basato sulla cura dell'individuo, si rende necessario alla luce dell'invecchiamento della popolazione generale e l'aumento di malattie legate all'età anziana [1,4]. Il Piano Nazionale di Ripresa e Resilienza (PNRR), approvato nel 2021, nel contesto del programma Europeo Next Generation EU, per quello che riguarda il sistema sanitario nazionale, ha da una parte l'obiettivo di modernizzare il paese, specialmente favorire la sanità digitale e lo sviluppo tecnologico, e dall'altra di favorire strategie per la diagnosi precoce e la prevenzione delle malattie [5,6].

Le malattie cardiovascolari (CardioVascular Disease, CVD) rappresentano la principale causa di mortalità e morbilità in Europa, e sono dovute alla somma di comportamenti di vita scorretti, fattori ambientali e predisposizione genetica [7]. Le varianti genetiche associate all'insorgenza di malattie cardiovascolari sono numerose, e il loro impatto è misurabile attraverso il Polygenic Risk Score (PRS), che attraverso una somma pesata dell'influenza di ogni singola variante sono in grado di stimare la predisposizione di ciascun individuo a sviluppare la malattia. Attraverso questi score, è possibile mettere in atto strategie preventive personalizzate, fatte su misura per il profilo di rischio di ogni paziente, per ritardarne o evitarne l'insorgenza. [8,9,10,11,12].

Nelle ultime due decadi, la prevalenza dei fattori di rischio per le malattie cardiovascolari è aumentata in maniera significativa. Diete poco salutari, inattività fisica e il fumo di sigaretta, risultano particolarmente elevati nei giovani nella fascia d'età 18-35, predisponendo allo sviluppo di patologie cardiovascolari [13], con conseguenze drammatiche sul futuro del sistema sanitario nazionale. Gli ultimi dati prevedono un incremento per il 2030 del 20% per quello che riguarda la mortalità, rispetto ai dati del 2019, e un aumento dei DALY (Disability Adjusted Life Years) [14].

Alla luce di questa epidemia di malattie cardiovascolari, la prevenzione diventa un obiettivo fondamentale per i sistemi sanitari globali. È ben noto che il miglioramento delle proprie abitudini alimentari, l'incremento dell'attività fisica e la cessazione dell'abitudine al fumo di sigaretta determinino una riduzione del rischio di sviluppare malattie cardiovascolari, e di conseguenza portino ad un diminuito burden in una fascia di età più avanzata. [15,16]

L'obiettivo di questo studio è di incentivare uno stile di vita salutare in soggetti sani, a basso rischio cardiovascolare tradizionale, attraverso la comunicazione del proprio profilo di rischio di predisposizione genetica, misurato attraverso il calcolo del PRS. Questo studio permetterebbe di valutare approcci innovativi personalizzati, con l'obiettivo ultimo di ridurre il carico di malattia cardiovascolare futura e i costi ad esso associati.

OBIETTIVI

Obiettivo generale

L'obiettivo generale dello studio è la valutazione della variazione nello stile di vita dopo la comunicazione del Polygenic Risk Score, misurato attraverso un questionario validato.

Obiettivo primario

- Valutare l'efficacia della comunicazione del Poligenic Risk Score nel modificare lo stile di vita dei partecipanti.

Obiettivo secondario

- Valutare la fattibilità dell'introduzione del Polygenic Risk Score nel percorso di cura del paziente

ENDPOINTS

Endpoint primario

- Variazione pre-post del punteggio dello stile di vita, misurato attraverso il Life Essentials' 8, a livello basale e al follow-up finale [17,18,19,20]

- Modifiche del profilo di rischio cardiovascolare valutate tramite SCORE2 / SCORE2 OP

Endpoints secondari

- Numero di partecipanti che hanno smesso o ridotto il fumo al follow-up finale.
- Numero di partecipanti che modificano il proprio modello alimentare al follow-up finale.
- Numero di partecipanti che smettono o riducono il consumo di alcol al follow-up finale.
- Numero di partecipanti che incrementano il proprio punteggio di attività fisica
- Accettabilità del test del Polygenic Risk Score per i pazienti
- Accettabilità del percorso sia per l'ospedale che per i pazienti.
- Compliance allo studio, misurata attraverso la percentuale di soggetti che completano tutti i follow-up
- Compliance agli interventi, misurata attraverso il numero di soggetti che aderiscono a frequenza, contenuti e durata dell'interventi proposto

METODI

Disegno dello studio

Studio sperimentale monocentrico, a singolo braccio, condotto su una coorte di soggetti afferenti al Policlinico Universitario A. Gemelli IRCCS di Roma (Italia), arruolati presso gli ambulatori del reparto di Cardiologia.

Popolazione

I partecipanti saranno selezionati tra i soggetti afferenti al Policlinico Universitario A. Gemelli IRCCS di Roma (Italia).

Il reclutamento verrà svolto presso gli ambulatori della prevenzione cardiovascolare, in collaborazione con la UOC Cardiologia.

Durata dello studio

7 mesi

Criteri di inclusione

- Rischio cardiovascolare tradizionale, misurato con lo SCORE 2 (Rischio basso (< 2,5%); Rischio moderato (tra 2,5% e 5%); Rischio elevato (tra 5% e 10%) oppure con lo SCORE 2-OP (Rischio moderato (< 7,5%); Rischio elevato (tra 7,5% e 15%)
- Disponibilità di esami ematochimici entro i 6 mesi precedenti
- Soggetti con età ≥ 40 anni.

Criteri di esclusione

- rischio cardiovascolare molto elevato misurato con lo SCORE 2 (rischio molto elevato > 10%) oppure con SCORE 2-OP (rischio molto elevato > 15%)
- Diabete
- Ipercolesterolemia familiare
- Pregressi eventi cardiovascolari

Procedure

Lo studio prevede una fase di arruolamento iniziale (T0) rivolta a soggetti afferenti al Policlinico Universitario A. Gemelli IRCCS di Roma (Italia).

Ai soggetti che accetteranno di partecipare allo studio PHC, verrà somministrato il consenso informato specifico per questo studio e verrà eseguita una valutazione basale, comprensiva dei questionari da compilare e il prelievo ematico per la valutazione del PRS. Il risultato della valutazione

del PRS verrà comunicato in una visita divulgativa circa un mese dopo (T1). I soggetti, infine, verranno rivalutati a sei mesi (T2) dalla visita divulgativa per la rivalutazione del proprio stile di vita.

In dettaglio, al momento dell'arruolamento (T0) tutti i partecipanti firmeranno il consenso informato e saranno sottoposti a una valutazione completa:

- compileranno il questionario LE’8, comprendente informazioni su stato socioeconomico e stili di vita, in particolare lo stato di fumatore, il consumo di alcol, il modello alimentare, il modello di sonno e l'attività fisica.

- saranno sottoposti a una visita medica completa per rilevare i dati biometrici (come BMI, circonferenze corporee), la frequenza cardiaca e la pressione sanguigna.

- i pazienti forniranno analisi del sangue eseguite autonomamente negli ultimi sei mesi, riportanti i valori del profilo lipidico (colesterolo totale, colesterolo HDL e LDL, trigliceridi), della glicemica o dell’emoglobina glicata, per effettuare il calcolo del punteggio SCORE-2 / SCORE 2- OP. Qualora tali analisi risultassero mancanti o troppo datate, onde evitare di escludere il paziente, si procederà con la raccolta di un campione ematico per la determinazione dei valori necessari sopracitati.

- prelievo ematico per analisi e calcolo del profilo di predisposizione genetica a sviluppare malattie cardiovascolari, attraverso il Polygenic Risk Score.
- è prevista la conservazione di campioni biologici (sangue intero).

Per ciascun partecipante verrà calcolato un punteggio relativo allo stile di vita, sulla base dello score del LE8.

Durante la visita telematica divulgativa (T1) verrà spiegato il punteggio relativo allo stile di vita. I partecipanti riceveranno consigli preventivi personalizzati scritti per ridurre i comportamenti scorretti e modificare lo stile di vita. Saranno inoltre resi noti i risultati del PRS a tutti i partecipanti e verrà fornita la categoria del profilo di rischio, con tutte le informazioni al riguardo. Al follow-up a sei mesi da T1 (T2), i partecipanti riceveranno una valutazione finale completa, come a T0, che

prevede la sottomissione del questionario sullo stile di vita e il ricalcolo della categoria dello stile di vita, e gli esami biometrici (peso, BMI, circonferenze corporee, frequenza cardiaca, pressione sanguigna). Contestualmente, per una nuova attribuzione dello SCORE2/SCORE2 OP, si procederà con un prelievo per la rivalutazione del profilo lipidico (colesterolo totale, HDL, LDL), della glicemia/emoglobina glicata. Inoltre verrà effettuato il dosaggio della lipoproteina (a) e della troponina I.

RACCOLTA, REGISTRAZIONE ED ANALISI STATISTICA DEI DATI

Questionario

Il questionario dello studio si articola in diverse sezioni.

Il questionario sullo stile di vita (Life's Essential 8) verrà somministrato a T0 e T2, e dopo compilazione, fornirà un punteggio relativo allo stile di vita che classificherà il paziente in una di tre categorie (favorevole, intermedio, sfavorevole). Il questionario è stato validato su popolazione europea e già utilizzato in diversi studi clinici. La sua scala va da 0 a 100 [20]. È suddiviso in diverse sezioni, articolate come segue:
Stile di vita generale: Abitudine al fumo, consumo di alcol, attività fisica, e quantità di sonno
Alimentazione: Tipologia di alimenti inclusi nella propria dieta
Fattori di salute: BMI, colesterolo, pressione sanguigna e glicemia

Questionario sulla storia personale e familiare, status socioeconomico e professionale e le informazioni demografiche, somministrato a T0.

Questionario riguardante l'accettabilità dell'intervento a medici e pazienti, somministrato a T2.

Questionario su valori e preferenze sull'utilizzo delle nuove tecnologie [21], somministrato a T2.

Questionario FACToR, ovvero una versione riadattata del questionario MICRA, per valutare la reazione al test genetico [22][23] somministrato a T2.

Un questionario per la valutazione dei livelli di ansia (GAD-7) [24], somministrato a T0 e a T2.

Interventi

- I campioni di sangue raccolti per il calcolo del profilo di predisposizione genetica verranno inviati alla Sezione di Igiene. In questa sede si procederà con l'estrazione del DNA e la

successiva genotipizzazione attraverso il macchinario GeneTitan™ MC Fast Scan Instrument, dell’azienda Thermo Fisher Scientific, e calcolo del PRS tramite Microarray.

I risultati consentiranno di stratificare gli individui in categorie di rischio cardiovascolare differenti

Conservazione Materiale Biologico

È prevista la raccolta di campioni ematici (sangue intero), che verranno riposti presso la Biobanca di FPG – Biobanca. Tali campioni potranno essere utilizzati per eventuali, ulteriori studi futuri previo consenso specifico.

Registrazione dei dati

I dati dello studio saranno raccolti e gestiti tramite e tramite REDCap, uno strumento elettronico di acquisizione dati presente presso FPG (https://redcap-irccs.policlinicogemelli.it/). REDCap (Research Electronic Data Capture) è una applicazione sicura basata sul Web progettata per supportare l'acquisizione di dati per studi di ricerca. Fornisce:

- Un’interfaccia intuitiva per l'immissione di dati convalidati;
- Un monitoraggio della manipolazione e dell'esportazione dei dati;
- Procedure di esportazione automatizzate per scaricare senza problemi i dati sui comuni pacchetti statistici**;**
- Procedure per l'importazione di dati da fonti esterne.

Tutte le soluzioni tecniche utili alla validazione di sistema saranno implementate con particolare attenzione all’integrità, alla consistenza ed alla completezza dei dati. Solo persone ufficialmente registrate come sperimentatori di studio o gestori di dati riceveranno un login utente per accedere alla piattaforma web ed inserire/gestire i dati. I dati, infine, verranno esportati in forma pseudoanonimizzata per l’analisi statistica.

CONFIDENZIALITÀ DELLE INFORMAZIONI

I dati personali dei pazienti arruolati saranno trattati in ottemperanza a quanto previsto dal D.lgs 196/03 e di tutta la normativa vigente. L'accesso ai campioni sarà limitato al responsabile della ricerca ed ai suoi collaboratori.

VALUTAZIONE DEL RISCHIO DI COERCIZIONE O INDEBITA INFLUENZA

Tutte le attività di studio e ricerca che coinvolgono i dipendenti saranno gestite rispettando la Procedura Aziendale 'Coinvolgimento dei dipendenti negli studi condotti in FPG' (PRO.1033), garantendo il corretto coinvolgimento e la tutela dei diritti dei lavoratori, nel rispetto delle normative in materia di privacy, sicurezza e benessere sul posto di lavoro.

ll colloquio informativo con i soggetti coinvolti sarà condotto in modo da garantire che i partecipanti abbiano sufficiente tempo e le condizioni adeguate per comprendere appieno lo studio, le sue finalità, i rischi e i benefici, e per esprimere il proprio consenso in piena libertà. Sarà assicurato che i partecipanti non subiscano alcuna forma di pressione o influenza indebita, e che possano prendere una decisione senza timore di ripercussioni professionali o personali.

Modalità di promozione e arruolamento dei partecipanti

La partecipazione allo studio sarà inoltre favorita attraverso azioni informative mirate, tra cui:

- La diffusione di materiale promozionale (es. locandine, brochure, infografiche)
- La condivisione di contenuti informativi sui canali digitali istituzionali e non (sito web, newsletter, social media)

Queste modalità sono finalizzate a garantire una comunicazione capillare ed efficace nei confronti del partecipante potenzialmente eleggibile per lo studio.

ANALISI STATISTICA

Calcolo del campione

Lo studio verrà proposto a tutti i dipendenti del Policlinico Gemelli che soddisfano i criteri di

inclusione prima riportati. Si stima che su un totale di circa 650 soggetti a cui verrà proposto l'intervento, il 70% aderirà al progetto (N=455) e di questi l'80% completerà lo studio (N=364). Tale dimensione del campione ci permette di rilevare con una potenza maggiore del 90% una differenza media di LE8 pari a 3 punti (deviazione standard=2) tra la fine e l'inizio del follow up.

Analisi Statistica

L'analisi statistica prevede l'applicazione di statistiche descrittive per descrivere il campione analizzato in termini di dati anagrafici, stili di vita, fattori di salute, parametri ematochimici raccolti al baseline e attitudini. Variazioni nello stile di vita verranno analizzati tramite modelli aggiustati ad effetto misto per misure ripetute. Verrà analizzato un potenziale elenco di moderatori e mediatori della relazione tra intervento e cambiamento comportamentale (come caratteristiche sociodemografiche, etnia, stato socioeconomico, istruzione, livelli di PRS) per valutare i meccanismi che spiegano il motivo per cui l'intervento può portare o meno al cambiamento, ovvero se persone con determinate caratteristiche possono beneficiarne più o meno. Le analisi statistiche saranno condotte utilizzando STATA (StataCorp, USA) e R.

REFERENCES

1. Beccia F. et al, An overview of Personalized Medicine landscape and policies in the European Union. European Journal of Public Health, 01 Nov 2022, 32(6):844-851 https://doi.org/10.1093/eurpub/ckac103 PMID: 36305782 PMCID: PMC9713394
2. PROPHET a PeRsOnalised Prevention roadmap for the future HEalThcare - https://prophetproject.eu/
3. EU Health Policy https://health.ec.europa.eu/eu-health-policy/overview_en
4. Personalised Medicine - European Commision (europa.eu) - Personalised medicine - European Commission (europa.eu)
5. PNRR – Governo Italiano Presidenza del Consiglio dei Ministri - PNRR_0.pdf (governo.it)
6. Next Generation Italia, approvato il piano del Governo - Next Generation Italia, approvato il piano del Governo (innovazione.gov.it)
7. About Cardiovascular Disease in ESC Member Countries; (Fact sheets for Press (escardio.org))
8. Knowles, J. W., Zarafshar, S., Pavlovic, A., Goldstein, B. A., Tsai, S., Li, J., McConnell, M. V., Absher, D., Ashley, E. A., Kiernan, M., Ioannidis, J. P. A., & Assimes, T. L. (2017). Impact of a Genetic Risk Score for Coronary Artery Disease on Reducing Cardiovascular Risk: A Pilot Randomized Controlled Study. Frontiers in cardiovascular medicine, 4, 53. https://doi.org/10.3389/fcvm.2017.00053
9. Widén, E., Junna, N., Ruotsalainen, S., Surakka, I., Mars, N., Ripatti, P., Partanen, J. J., Aro, J., Mustonen, P., Tuomi, T., Palotie, A., Salomaa, V., Kaprio, J., Partanen, J., Hotakainen, K., Pöllänen, P., & Ripatti, S. (2022). How Communicating Polygenic and Clinical Risk for Atherosclerotic Cardiovascular Disease Impacts Health Behavior: an Observational Follow-up Study. Circulation. ac and precision medicine, 15(2), e003459. https://doi.org/10.1161/CIRCGEN.121.003459
10. Fenton, G. L., Smit, A. K., Keogh, L., & Cust, A. E. (2019). Exploring the emotional and behavioural reactions to receiving personalized melanoma genomic risk information: a qualitative study. The British journal of dermatology, 180(6), 1390–1396. https://doi.org/10.1111/bjd.17582
11. Lambert, S. A., Abraham, G., & Inouye, M. (2019). Towards clinical utility of polygenic risk scores. *Human molecular genetics*, *28*(R2), R133–R142. https://doi.org/10.1093/hmg/ddz187
12. Lloyd-Jones DM, Allen NB, Anderson CAM, et al. Life's Essential 8: Updating and Enhancing the American Heart Association's Construct of Cardiovascular Health: A Presidential Advisory From the American Heart Association. Circulation. 2022;146(5):e18-e43. doi:10.1161/CIR.0000000000001078
13. Charlotte Andersson 1 2, Ramachandran S Vasan, Epidemiology of cardiovascular disease in young individuals, Nature Reviews Cardiology, 2018 Apr;15(4):230-240. doi: 10.1038/nrcardio.2017.154. Epub 2017 Oct 12. PMID: 29022571, DOI: 10.1038/nrcardio.2017.154 - https://pubmed.ncbi.nlm.nih.gov/29022571/
14. Wang H, Liu J, Feng Y, Ma A, Wang T. The burden of cardiovascular diseases attributable to metabolic risk factors and its change from 1990 to 2019: a systematic analysis and prediction. Front Epidemiol. 2023 May 25;3:1048515. doi: 10.3389/fepid.2023.1048515. PMID: 38455920; PMCID: PMC10910969.

15. Kiang Liu, Martha L. Daviglus, Catherine M. Loria, Laura A. Colangelo, Bonnie Spring, Arlen C. Moller, and Donald M. Lloyd-Jones, Healthy Lifestyle through Young Adulthood and Presence of Low Cardiovascular Disease Risk Profile in Middle Age: The Coronary Artery Risk Development in (Young) Adults (CARDIA) Study, Circulation. 2012 Feb 28; 125(8): 996–1004. doi: 10.1161/CIRCULATIONAHA.111.060681, PMID: 22291127, PMCID: PMC3353808
16. Charlotte Andersson & Ramachandran S. Vasan, Epidemiology of cardiovascular disease in young individuals, Nat Rev Cardiol 15, 230–240 (2018). https://doi.org/10.1038/nrcardio.2017.154
17. Petermann-Rocha F, Deo S, Celis-Morales C, et al. An Opportunity for Prevention: Associations Between the Life's Essential 8 Score and Cardiovascular Incidence Using Prospective Data from UK Biobank. *Curr Probl Cardiol*. 2023;48(4):101540. doi:10.1016/j.cpcardiol.2022.101540
18. He P, Zhang Y, Ye Z, et al. A healthy lifestyle, Life's Essential 8 scores and new-onset severe NAFLD: A prospective analysis in UK Biobank. *Metabolism*. 2023;146:155643. doi:10.1016/j.metabol.2023.155643
19. Zhang J, Chen G, Habudele Z, et al. Relation of Life's Essential 8 to the genetic predisposition for cardiovascular outcomes and all-cause mortality: results from a national prospective cohort. *Eur J Prev Cardiol*. 2023;30(15):1676-1685. doi:10.1093/eurjpc/zwad179
20. Isiozor NM, Kunutsor SK, Voutilainen A, Laukkanen JA. Life's Essential 8 and the risk of cardiovascular disease death and all-cause mortality in Finnish men. *Eur J Prev Cardiol*. 2023;30(8):658-667. doi:10.1093/eurjpc/zwad040
21. Rosenstock IM. The Health Belief Model and Preventive Health Behavior. Health Education Monographs. 1974;2(4):354-386. doi:10.1177/109019817400200405
22. Cella, David et al. "A brief assessment of concerns associated with genetic testing for cancer: the Multidimensional Impact of Cancer Risk Assessment (MICRA) questionnaire." *Health psychology : official journal of the Division of Health Psychology, American Psychological Association* vol. 21,6 (2002): 564-72.
23. Li, Meng et al. "The Feelings About genomiC Testing Results (FACToR) Questionnaire: Development and Preliminary Validation." *Journal of genetic counseling*vol. 28,2 (2019): 477-490. doi:10.1007/s10897-018-0286-9
24. Spitzer RL, Kroenke K, Williams JBW, Löwe B. A Brief Measure for Assessing Generalized Anxiety Disorder: The GAD-7. Arch Intern Med. 2006;166(10):1092–1097. doi:10.1001/archinte.166.10.1092)
