# Supplementary material for: Polygenic Risk Scores disclosure for cardiovascular prevention: Protocol of the Personalized HeartCare (PHC) trial
Source: PLoS One. 2026 Apr 6;21(4):e0345294. doi: 10.1371/journal.pone.0345294 (PMC13052841; doi:10.1371/journal.pone.0345294)
Supplement: S3 File — (PDF) [file pone.0345294.s003.pdf]

Supplementary File S1. Informed consent in the participant's in English language.

INFORMATION SHEET FOR PARTICIPATION IN A CLINICAL TRIAL AND CONSENT FORM
for an adult patient capable of giving consent personally

- TITLE: Personalised HeartCare (**PHC**): Innovative Approaches for Personalized Primary Prevention of Cardiovascular Diseases

DEPARTMENT/CLINIC/SERVICE:
Department of Women's, Children's, and Public Health Sciences PRINCIPAL INVESTIGATOR: Prof. Stefania Boccia
Phone: 0630154396

Dear Sir or Madam,
The information contained in the following information sheet is very detailed. We ask that you agree to participate in the trial ONLY after carefully reading this sheet and having a THOROUGH DISCUSSION with a member of the research team, who will take the TIME NECESSARY to ensure you fully understand what is being proposed. It is your right to be informed about the purpose and characteristics of the trial so that you can make an informed and voluntary decision about whether to participate.
The purpose of this document is to inform you about the nature of the clinical trial, its objectives, and what your participation will entail, including your rights and responsibilities. We encourage you to read the following information carefully. The researchers involved in this project, listed at the beginning of this document, are available to answer your questions. No question you may have is trivial: please do not hesitate to ask!
In addition to discussing the proposal in this document with us, you may also discuss it with your family doctor, your family members, and other people you trust. Please take all the time you need to decide. You may take home an unsigned copy of this document to think it over or discuss it with others before making a decision.
If you decide not to participate in the trial, you will still receive the best possible care for patients with your condition/disease.
Your refusal will in no way be interpreted as a lack of trust.

The Principal Investigator

INFORMATION SHEET

Dear Sir/Madam,

The A. Gemelli University Polyclinic-IRCCS Foundation is planning a study titled: *"Personalised HeartCare (**PHC**): innovative approaches for personalized primary prevention of cardiovascular diseases*."

This national, single-center study aims to evaluate whether communicating an individual's genetic predisposition to cardiovascular disease, using Polygenic Risk Scores (PRS), can positively influence lifestyle. Specifically, we want to determine whether this information can encourage changes in daily behaviors, promoting healthier choices. At the end of the study, a questionnaire will be administered to assess any changes in lifestyle, with the goal of contributing to the prevention of cardiovascular diseases.

To conduct this research, we would like to rely on the cooperation and willingness of people who, like you, meet the scientific criteria for the evaluation that will be carried out. Whether or not you decide to participate in this study will have no impact on the care you receive, and the doctors will continue to treat you with the utmost care.

Before you decide whether to accept or decline participation, however, please read these pages carefully, taking all the time you need, and ask for clarification if anything is unclear or if you need further explanation. Additionally, if you wish, you may consult with your family members or a trusted physician before making your decision.

WHAT THE STUDY AIMS TO DO

The study's main objective is to collect detailed information about your lifestyle, including dietary habits, physical activity levels, sleep quality, alcohol consumption, and smoking habits. Additionally, certain vital signs will be monitored to assess any overall improvements in lifestyle. The ultimate goal of the study is to examine potential changes in lifestyle categories using the validated **"LIFE'S ESSENTIAL** 8" questionnaire. Additionally, the study aims to analyze the feasibility and acceptability of an innovative intervention for the primary prevention of cardiovascular diseases, with the intent of providing useful guidance for the implementation of such interventions within the National Health System.

WHAT THE STUDY INVOLVES

The study design for this research involves you receiving personalized advice to improve your lifestyle, based on risk factors identified through a questionnaire. If you decide to participate, you will have an in-person visit during which you will receive medical recommendations for the prevention of cardiovascular disease. This advice will be tailored to your specific habits and risk factors, in accordance with European guidelines.

The visit will include a biometric examination to assess certain parameters, such as weight, BMI, body measurements, heart rate, and blood pressure. Additionally, you will be asked to provide a blood sample, which is necessary to generate your genetic predisposition profile by calculating your **Polygenic Risk Score**. This profile will provide further information about your cardiovascular risk. At the same time, if you have not already had them done, we will proceed with an assessment of your lipid profile (total cholesterol, HDL, LDL, triglycerides) and blood glucose/glycated hemoglobin. These assessments will be

These tests are also repeated during the final visit, along with measurements of lipoprotein A (LpA) and troponin I.

It is important to note that the study does not involve the administration of medications or the performance of invasive procedures.

WHAT YOUR PARTICIPATION IN THE STUDY ENTAILS

If you agree to participate in this study, you will undergo an initial visit to verify that your condition meets the criteria for participation.
You will be provided with a complete and detailed description of the project, and only then, if you decide to proceed, will you be asked to sign the informed consent form.

The cooperation we are requesting from you consists of:

- Providing a blood sample for the assessment of the Polygenic Risk Score (PRS) and, if valid blood chemistry tests are not available, we will also assess your lipid profile (total cholesterol, HDL, LDL, triglycerides) and blood glucose (or, alternatively, glycated hemoglobin).

- Undergoing a biometric examination to collect certain data/information (weight, BMI, body measurements, heart rate, and blood pressure)

- Complete a questionnaire that will gather information on lifestyle, socioeconomic status, area of residence, and various health-related behaviors, including smoking, alcohol consumption, dietary habits, sleep patterns, and physical activity. This questionnaire will be administered again approximately 7 months later to assess any changes.

The results of the analysis will be discussed with you during an informational visit that will take place approximately four weeks after you enroll in the study. At that time, you will be provided with your genetic predisposition profile.
In addition, a second visit is scheduled after seven months, during which you will be asked to complete the questionnaire again and provide a blood sample for reassessment of your lipid profile (total cholesterol, HDL, LDL, and triglycerides), blood glucose (or glycated hemoglobin), lipoprotein
(a), and troponin I

The study will last 7 months and will involve 455 participants from the A. Gemelli IRCCS University Hospital in Rome (Italy).

Participation in the study involves no financial cost to you, nor does it provide any compensation.

WHAT ARE THE RISKS ASSOCIATED WITH PARTICIPATING IN THE STUDY

Participation in the study is not expected to involve any risks associated with the administration of medications or treatments.

WHAT BENEFITS CAN YOU EXPECT FROM PARTICIPATING IN THE STUDY?

Participation in this study does not provide any immediate direct benefits for you. However, for participants who adopt a healthier lifestyle, participation in the study could lead to a reduction in cardiovascular risk. Furthermore, the study will allow us to better understand the impact of new technologies on the primary prevention of cardiovascular events. Your participation will therefore contribute to scientific progress and may have a positive impact on other patients in the future.

STUDY RESULTS AND CONFIDENTIALITY OF COLLECTED INFORMATION

All of your data and biological samples will be pseudonymized; that is, you will be assigned a code that cannot be directly linked to you, and they will be recorded electronically in the RedCap system to create the database. This code will not allow you to be identified outside the medical treatment center.

With regard to the processing of personal data, please refer to the specific privacy notice regarding consent to the processing of personal data, which will be provided to you at the same time on a separate sheet.

WHAT HAPPENS IF YOU DECIDE NOT TO PARTICIPATE IN THE STUDY—POSSIBLE ALTERNATIVE TREATMENTS.

You are free to choose not to participate in the study. In this case, the doctors will continue to care for you with the same level of attention.

HOW WILL MY BIOLOGICAL SAMPLES COLLECTED FOR THE PURPOSES OF THE TRIAL BE PROCESSED, AND WHO WILL HAVE ACCESS TO THEM?

As with your health data, your pseudo-anonymized biological samples will also be
used for the purposes of the trial. Specifically, blood samples collected from all participants for the purpose of analyzing cardiovascular genetic risk will be processed at the Department of Hygiene of the Biological Institutes of the Catholic University of the Sacred Heart. Additionally, the collection and storage of blood samples (whole blood) are planned.

WHAT HAPPENS IN THE EVENT OF HARM

The institutional policy applies to this study.

HOW WILL MY BIOLOGICAL SAMPLES COLLECTED FOR THE PURPOSES OF THE TRIAL BE HANDLED, AND WHO WILL HAVE ACCESS TO THEM?

As with your health data, your pseudo-anonymized biological samples will also be used
for the purposes of the trial.
In addition, the collected blood samples (whole blood) will be stored at the FPG Biobank. These samples may be used for any future studies.

WITHDRAWAL FROM THE STUDY

Your participation in this research program is entirely voluntary, and you may withdraw from the study at any time by notifying the Investigator: Prof. Stefania Boccia. In this case, the data collected up to the time of withdrawal will be included in the results in an aggregated and anonymous form for the final analysis.

Similarly, the trial may be discontinued if:

1. The physician does not observe any benefit, or if adverse effects or other issues arise;
2. If new information becomes available and participation in the trial is no longer in your best interest;
3. You do not follow the agreed-upon rules for participating in the trial;
4. The trial is discontinued by the regulatory authorities or the sponsor.

If data becomes available that could influence the decision to continue the study in question, you will be promptly informed. If you decide to continue the study, you may be asked to sign a new informed consent form highlighting the update.

INFORMATION ABOUT THE STUDY RESULTS

If you request it, at the end of the study you may be informed of the study results in general and, specifically, those that pertain to you.

FURTHER INFORMATION

For further information and updates during the study, please contact the following staff members:

- Prof. Stefania Boccia
  Tel. 0630154396
  Email: stefania.boccia@unicatt.it

If you deem it necessary, you may inform your family doctor about your participation in this study.

The study protocol proposed to you has been drafted in accordance with the current revisions of the European Union's Good Clinical Practice Guidelines and the World Medical Association's Declaration of Helsinki on clinical trials involving human subjects, and has been approved by the Lazio Area 3 Regional Ethics Committee. You may report any matter you deem appropriate to highlight regarding the trial in which you are participating to the Ethics Committee, the Health Directorate of this facility, and the Competent Authority (AIFA).

WHO IS ORGANIZING AND SPONSORING THIS STUDY?

This study is sponsored by the Catholic University of the Sacred Heart in Rome
Thank you for your attention and for taking the time to read and discuss this document.
If you decide to participate in the study, you will be provided with a copy of this Information Sheet and a signed Consent Form to keep.

CONSENT FORM

(*This statement must be personally signed and dated by the patient and by the physician who conducted the informed consent discussion*)

I DECLARE

○ that I have received from Dr. ____
exhaustive explanations regarding the request to participate in the research in question, as set forth in the information section of which I was given a copy earlier, which forms part of this consent, and of which I was given a copy on ____________________________________

○ that the nature, purposes, procedures, expected benefits, possible risks and disadvantages, and alternative treatment options to the proposed clinical trial have been clearly explained to me and I have understood them;

○ that I have had the opportunity to ask the study investigator any questions and have received satisfactory answers;

○ that I have had sufficient time to reflect on the information received;

○ that I had sufficient time to discuss this with third parties;

○ that I have been informed that the trial protocol and all forms used have been approved by the competent Ethics Committee;

○ that I am aware the study may be discontinued at any time;

○ that I have been informed that I will be notified of any new information that could compromise the safety of the research and that, for any problems or further questions, I may contact the principal investigator or his/her staff;

○ that, for the best protection of my health, I am aware of the importance of informing my primary care physician about the trial in which I agree to participate.

○ I am aware of the importance of providing the investigator with all relevant information (medications, side effects, etc.) concerning me;

○ that I have been informed that the results of the study will be disclosed to the scientific community, while protecting my identity in accordance with current privacy regulations;

○ that I am aware that any choice expressed in this consent form may be revoked at any time and without any justification;

○ that I have received a copy of this consent form.

Place and date ____

____
Patient's First Name and Last Name (in block letters)

Participant's signature

(*If the participant is unable to read or sign, a witness who is independent of the investigator and the sponsor must be present throughout the entire informed consent discussion. The witness must personally sign and date the informed consent form after the form itself and any other written information have been read and explained to the subject and the subject has given verbal consent to participate in the study*).

In this case:

I the undersigned ………………………………. certify that the Dr. ……………………………………………………………has thoroughly explained to Mr. ……………………………………………………………
the details of the clinical trial in question, as described in the attached information sheet, and that, having had the opportunity to ask all the questions he deemed necessary, he freely agreed to participate in the trial.

Date……………. Signature of the independent witness …………………………………….

Date… ....Signature of the physician who provided the information to the patient ………………………………

STATEMENT BY THE PHYSICIAN WHO OBTAINED CONSENT

I, the undersigned (FIRST NAME-LAST

NAME), in my capacity as
○ Principal Investigator

○ Delegate of the Principal

Investigator DECLARE

that the Patient has voluntarily consented to participate in the trial

I further declare that:
○ have provided the patient with a thorough explanation of the purpose of the trial, the procedures, the potential risks and benefits, and possible alternatives;

○ verified that the Patient has sufficiently understood the information provided to him or her;

○ to have given the Patient sufficient time and the opportunity to ask questions regarding the trial;

○ to have clearly explained the possibility of withdrawing from the trial at any time or of changing the choices made;

○ not to have exercised any coercion or undue influence in requesting this consent;

○ have provided the patient with information on how the trial results will be communicated to him or her.

____ ________

Place and date ____

____
First Name Last Name (in block letters) of the physician who provided the information and obtained consent

Signature (and stamp)

This form is an integral part of the informed consent form and must be kept together with it

Supplementary File S1. Informed consent in native language.

FOGLIO INFORMATIVO PER LA PARTECIPAZIONE AD UNA SPERIMENTAZIONE CLINICA E DICHIARAZIONE DI CONSENSO

per un paziente adulto capace di dare personalmente il consenso

- TITOLO: Personalised HeartCare (**PHC**): approcci innovativi per la prevenzione primaria personalizzata delle malattie cardiovascolare

REPARTO/AMBULATORIO/SERVIZIO:
Dipartimento di scienze della salute della donna, del bambino e di sanità pubblica
SPERIMENTATORE: Prof.ssa Stefania Boccia
Telefono: 0630154396

Gent.le Sig.ra/re,
le informazioni contenute nel seguente foglio informativo sono molto dettagliate. Le chiediamo di accettare di partecipare alla sperimentazione SOLO dopo aver letto con attenzione questo foglio ed avere avuto un COLLOQUIO ESAURIENTE con un componente del gruppo di sperimentazione che le dovrà dedicare il TEMPO NECESSARIO per comprendere completamente ciò che le viene proposto.
È Suo diritto essere informata/o circa lo scopo e le caratteristiche della sperimentazione affinché Lei possa decidere in modo consapevole e libero se partecipare.
Questo documento ha l'obiettivo di informarLa sulla natura della sperimentazione, sul fine che esso si propone, su ciò che comporterà per Lei la partecipazione, compresi i suoi diritti e responsabilità.
La invitiamo a leggere attentamente quanto riportato di seguito. I ricercatori coinvolti in questo progetto, indicati all'inizio di questo documento, sono a disposizione per rispondere alle Sue domande. Nessuna domanda che Le viene in mente è banale: non abbia timore di farla!
Oltre che con noi può discutere la proposta contenuta in questo documento con il Suo medico di famiglia, i Suoi familiari e altre persone di sua fiducia. Si prenda tutto il tempo necessario per decidere. Può portare a casa una copia non firmata di questo documento per pensarci o per discuterne con altri prima di prendere una decisione.
Se decide di non partecipare alla sperimentazione, riceverà comunque la migliore assistenza possibile per i pazienti con la Sua condizione/malattia.
Un Suo rifiuto non sarà in alcun modo interpretato come una mancanza di fiducia.

Lo Sperimentatore Principale

SCHEDA INFORMATIVA

Gentile Signora/Gentile Signore,

Presso la Fondazione Policlinico Universitario A. Gemelli-IRCCS è in programma uno studio dal titolo: "*Personalised HeartCare (**PHC**): approcci innovativi per la prevenzione primaria personalizzata delle malattie cardiovascolari*".

Questa ricerca, di carattere nazionale e monocentrica, si propone di valutare se la comunicazione della predisposizione genetica individuale alle malattie cardiovascolari, attraverso l'uso dei Polygenic Risk Scores (PRS), possa influenzare positivamente lo stile di vita. In particolare, vogliamo verificare se questa informazione possa stimolare cambiamenti nei comportamenti quotidiani, favorendo scelte più salutari. Alla fine dello studio, un questionario sarà somministrato per rilevare eventuali modifiche nello stile di vita, con l'obiettivo di contribuire alla prevenzione delle malattie cardiovascolari.

Per svolgere tale ricerca vorremmo avvalerci della collaborazione e della disponibilità di persone che, come Lei, soddisfino i requisiti scientifici idonei alla valutazione che verrà eseguita. Il fatto di decidere o meno di partecipare a questo studio non avrà alcun impatto sulla assistenza che riceverà ed i medici continueranno a seguirLa con la dovuta attenzione.

Prima, però, che Lei prenda la decisione di accettare o rifiutare di partecipare, La preghiamo di leggere con attenzione queste pagine, prendendo tutto il tempo che Le necessita, e di chiedere chiarimenti qualora non avesse ben compreso o avesse bisogno di ulteriori precisazioni. Inoltre, qualora lo desiderasse, prima di decidere può chiedere un parere ai Suoi familiari o ad un Suo medico di fiducia.

CHE COSA SI PROPONE LO STUDIO

Lo studio ha come obiettivo principale la raccolta di informazioni dettagliate sul Suo stile di vita, comprendendo abitudini alimentari, livelli di attività fisica, qualità del sonno, consumo di alcol e abitudini legate al fumo. Inoltre, verranno monitorati alcuni parametri vitali per valutare eventuali miglioramenti complessivi nello stile di vita. L'obiettivo finale dello studio è esaminare eventuali cambiamenti nella categoria dello stile di vita, utilizzando il questionario validato **"LIFE'S ESSENTIAL 8"**. Secondariamente, lo studio si propone di analizzare la fattibilità e l'accettabilità di un intervento innovativo per la prevenzione primaria delle malattie cardiovascolari, con l'intento di fornire indicazioni utili per l'implementazione di tali interventi nel Sistema Sanitario Nazionale.

COSA COMPORTA LO STUDIO

Il disegno sperimentale di questa ricerca prevede che Lei riceva consigli personalizzati per migliorare il Suo stile di vita, in base ai fattori di rischio identificati attraverso un questionario. Se decide di partecipare, avrà una visita in persona durante la quale Le verranno forniti suggerimenti medici per la prevenzione delle malattie cardiovascolari. Questi consigli saranno adattati alle Sue specifiche abitudini e ai Suoi fattori di rischio, seguendo le linee guida europee.

La visita includerà un esame biometrico per la valutazione di alcuni parametri, come peso, BMI, circonferenze corporee, frequenza cardiaca e pressione sanguigna. Inoltre, Le sarà richiesto di sottoporsi a un prelievo ematico, necessario per elaborare il Suo profilo di predisposizione genetica attraverso il calcolo del **Polygenic Risk Score**. Questo profilo fornirà ulteriori informazioni sul Suo rischio cardiovascolare. Contestualmente, qualora ne fosse sprovvisto, si procederà con la valutazione del profilo lipidico (colesterolo totale, HDL, LDL, trigliceridi) e glicemia/emoglobina glicata. Queste valutazioni verranno

inoltre ripetute in occasione della visita conclusiva, insieme alla valutazione della lipoproteinaA (lpA) ed alla troponina I.

È importante sottolineare che lo studio non prevede la somministrazione di farmaci né l'esecuzione di indagini invasive.

COSA COMPORTA LA SUA PARTECIPAZIONE ALLO STUDIO

Se accetta di partecipare a questo studio Lei verrà sottoposto/a ad una prima visita per verificare che le sue condizioni soddisfino i criteri richiesti per la partecipazione.
Le verrà fornita una descrizione completa e dettagliata del progetto e, solo successivamente, se deciderà di procedere, Le sarà richiesto di firmare il consenso informato.

La collaborazione che Le viene richiesta consiste nel:

- Effettuare un prelievo ematico, per la valutazione del Polygenic Risk Score (PRS) ed eventualmente, se sprovvisto di analisi ematochimiche valide, si provvederà alla valutazione del profilo lipidico (colesterolo totale, HDL, LDL, trigliceridi) e glicemia (in alternativa emoglobina glicata).

- Sottoporsi ad un esame biometrico per ricavare alcuni dati/informazioni (peso, BMI, circonferenze corporee, frequenza cardiaca e pressione sanguigna)

- Compilare un questionario che raccoglierà informazioni sullo stile di vita, lo stato socioeconomico, l'area di residenza e vari comportamenti legati alla salute, tra cui il fumo, il consumo di alcol, le abitudini alimentari, il modello di sonno e l'attività fisica. Questo questionario sarà somministrato nuovamente a distanza di circa 7 mesi per valutare eventuali cambiamenti.

Il risultato dell'analisi Le sarà comunicato durante una visita divulgativa che avverrà circa quattro settimane dopo l'arruolamento nello studio. In quella stessa occasione, Le verrà fornito il Suo profilo di predisposizione genetica.
Inoltre, è previsto un secondo incontro dopo sette mesi, durante il quale Le sarà richiesto di ricompilare il questionario ed effettuare un prelievo ematico per la rivalutazione del profilo lipidico (colesterolo totale, HDL, LDL e trigliceridi), glicemia (in alternativa emoglobina glicata), lipoproteina (a) e troponina I

Lo studio durerà 7 mesi e coinvolgerà 455 soggetti afferenti al Policlinico Universitario A. Gemelli IRCCS di Roma (Italia) .

La partecipazione allo studio non comporta alcun onere economico per Lei, né prevede alcun compenso.

QUALI SONO I RISCHI DERIVANTI DALLA PARTECIPAZIONE ALLO STUDIO

La partecipazione allo studio non comporta prevedibilmente nessun rischio legato alla somministrazione di farmaci/trattamenti.

QUALI SONO I BENEFICI CHE POTRÀ RICEVERE PARTECIPANDO ALLO STUDIO

La partecipazione a questo studio non comporta benefici diretti immediati per Lei. Tuttavia, per i partecipanti che adotteranno un miglioramento dello stile di vita, l'adesione allo studio potrebbe portare a una riduzione del rischio cardiovascolare. Inoltre, lo studio ci permetterà di comprendere meglio l'impatto delle nuove tecnologie nella prevenzione primaria degli eventi cardiovascolari. La Sua partecipazione contribuirà, quindi, al progresso della scienza e potrà avere un impatto positivo per altri pazienti in futuro.

RISULTATI DELLO STUDIO E RISERVATEZZA DELLE INFORMAZIONI RACCOLTE

Tutti i Suoi dati ed i campioni biologici saranno pseudo-anonimizzati, ossia Le sarà attribuito un codice non direttamente riconducibile alla sua persona e saranno registrati in formato elettronico nel sistema RedCap, per la creazione del database. Questo codice non consentirà di identificarLa all'esterno del centro medico del trattamento.

Per quanto riguarda il trattamento dei dati personali dovrà fare riferimento all'informativa specifica per la manifestazione del consenso al trattamento dei dati personali che Le verrà consegnata contestualmente, su foglio a parte.

COSA SUCCEDE SE DECIDE DI NON PARTECIPARE ALLO STUDIO-POSSIBILI TRATTAMENTI ALTERNATIVI.

Lei è libero di non partecipare allo studio. In questo caso, i medici continueranno a seguirla comunque con la dovuta attenzione assistenziale.

COME VERRANNO TRATTATI E CHI AVRÀ ACCESSO AI MIEI CAMPIONI BIOLOGICI PRELEVATI AI FINI DELLA SPERIMENTAZIONE

Come per i Suoi dati sanitari, anche i Suoi campioni biologici pseudo-anonimizzati, saranno utilizzati ai fini della sperimentazione. In particolare, i campioni di sangue prelevati da tutti i partecipanti con il fine di effettuare l'analisi del rischio genetico cardiovascolare, verranno lavorati presso la sezione di Igiene degli istituti Biologici dell'Università Cattolica del Sacro Cuore. Inoltre, è prevista la raccolta e conservazione di campioni ematici (sangue intero).

COSA SUCCEDE IN CASO DI DANNO

Per questo studio vige la polizza istituzionale.

COME VERRANNO TRATTATI E CHI AVRÀ ACCESSO AI MIEI CAMPIONI BIOLOGICI PRELEVATI AI FINI DELLA SPERIMENTAZIONE

Come per i Suoi dati sanitari, anche i Suoi campioni biologici pseudo-anonimizzati, saranno utilizzati ai fini della sperimentazione.

Inoltre, i campioni ematici raccolti (sangue intero) verranno riposti presso la Biobanca di FPG – Biobanca. Tali campioni potranno essere utilizzati per eventuali, ulteriori studi futuri.

INTERRUZIONE DELLO STUDIO

La Sua adesione a questo programma di ricerca è completamente volontaria e Lei potrà ritirare la sua partecipazione allo studio in qualsiasi momento dandone comunicazione allo Sperimentatore: Prof.ssa Stefania Boccia. In questo caso, i dati raccolti fino al momento del ritiro saranno considerati nei risultati in forma aggregata ed anonima per l'analisi finale.
Allo stesso modo, la sperimentazione potrà essere interrotta se:

1. Il medico non constaterà un giovamento oppure se saranno intervenuti effetti non desiderati o altro;
2. Si rendessero disponibili nuove informazioni e la sperimentazione non fosse più nel Suo migliore interesse;
3. Lei non seguisse le regole concordate per la partecipazione alla sperimentazione;
4. La sperimentazione venisse interrotta dalle autorità componenti o dal promotore.

Qualora divengano disponibili dati che possano influenzare la decisione di continuare lo studio in oggetto, sarà tempestivamente informato/a. Se Lei dovesse decidere di continuare lo studio, potrebbe venirLe chiesto di firmare un nuovo consenso informato in cui sarà evidenziato l'aggiornamento.

INFORMAZIONI CIRCA I RISULTATI DELLO STUDIO

Se Lei lo richiederà, alla fine dello studio potranno esserLe comunicati i risultati dello studio in generale ed in particolare quelli che La riguardano.

ULTERIORI INFORMAZIONI

Per ulteriori informazioni e comunicazioni durante lo studio potrà contattare il seguente personale:

- Prof.ssa Stefania Boccia
  Tel.0630154396
  e-mail: stefania.boccia@unicatt.it

Se Lei lo ritenesse necessario, potrebbe informare il Suo medico di famiglia circa la partecipazione a questo studio.

Il protocollo dello studio che Le è stato proposto è stato redatto in conformità alle revisioni correnti delle Norme di Buona Pratica Clinica della Unione Europea e della Dichiarazione di Helsinki della Associazione Medica Mondiale sugli studi clinici che interessano soggetti umani ed è stato approvato dal Comitato Etico Territoriale Lazio Area3 . Lei può segnalare qualsiasi fatto ritenga opportuno evidenziare, relativamente alla sperimentazione che La riguarda, al Comitato Etico e alla Direzione Sanitaria di questa struttura e all'Autorità Competente (AIFA).

CHI ORGANIZZA E PROMUOVE QUESTO STUDIO?

Lo studio è promosso dal Università cattolica del Sacro Cuore di Roma

La ringraziamo per l'attenzione e il tempo che ha dedicato alla lettura e alla discussione del presente documento.

Qualora Lei decidesse di partecipare allo Studio, le saranno forniti una copia di questa Scheda Informativa e un Modulo di Consenso sottoscritto da conservare.

DICHIARAZIONE DI CONSENSO

(*questa dichiarazione deve essere firmata e datata personalmente dal paziente e dal medico che ha condotto la discussione relativa al consenso informato*)

DICHIARO

○ di aver ricevuto dal Dottor ____ esaurienti spiegazioni in merito alla richiesta di partecipazione alla ricerca in oggetto, secondo quanto riportato nella sezione informativa della quale mi è stata data prima d'ora una copia, facente parte di questo consenso, della quale mi è stata consegnata una copia in data _____________________

○ che mi sono stati chiaramente spiegati ed ho compreso la natura, le finalità, le procedure, i benefici attesi, i rischi e gli inconvenienti possibili e le modalità di trattamento alternativo rispetto alla sperimentazione clinica proposta;

○ di aver avuto l'opportunità di porre qualsivoglia domanda allo sperimentatore dello studio e di aver avuto risposte soddisfacenti;

○ di aver avuto il tempo sufficiente per riflettere sulle informazioni ricevute;

○ di avere avuto il tempo sufficiente per discuterne con terzi;

○ di essere stato informato che il protocollo della sperimentazione e tutti i moduli utilizzati hanno avuto il parere favorevole del CET competente;

○ di essere consapevole che la ricerca potrà essere interrotta in ogni momento;

○ di essere stato informato che sarò messo al corrente di qualsiasi nuovo dato che possa compromettere la sicurezza della ricerca e che, per ogni problema o per ulteriori domande, potrò rivolgermi allo sperimentatore principale o ai suoi collaboratori;

○ che per la migliore tutela della mia salute sono consapevole dell'importanza di informare il medico di medicina generale della sperimentazione alla quale accetto di partecipare.

○ sono consapevole dell'importanza di fornire tutte le informazioni (farmaci, effetti collaterali, ecc.) che mi riguardano, allo sperimentatore;

○ di essere stato informato che i risultati dello studio saranno resi noti alla comunità scientifica, tutelando la mia identità secondo la normativa vigente sulla privacy;

○ di essere consapevole che qualsiasi scelta espressa in questo modulo di consenso potrà essere revocata in qualsiasi momento e senza alcuna giustificazione;

○ di aver ricevuto una copia del presente modulo di consenso.

Luogo e data ____

____
Nome Cognome del paziente (stampatello)

Firma del partecipante

(*Se il partecipante non è in grado di leggere o di firmare, un testimone indipendente dallo sperimentatore e dallo sponsor deve essere presente durante l'intera discussione relativa al consenso informato. Il testimone deve firmare e datare personalmente la dichiarazione di consenso informato dopo che il modulo stesso e qualsiasi altra informazione scritta siano stati letti e spiegati al soggetto e questi abbia espresso il consenso verbale alla partecipazione allo studio*).

In questo caso:

Io sottoscritto ………………………………. testimonio che il dottor ……………………………………………………………ha esaurientemente spiegato al Sig. …………………………………………………………….
le caratteristiche dello studio sperimentale in oggetto, secondo quanto riportato nella scheda informativa qui allegata, e che lo stesso, avendo avuto la possibilità di fare tutte le domande che ha ritenuto necessarie, ha accettato liberamente di aderire allo studio.

Data…………… Firma del testimone indipendente …………………………………..

Data…………… Firma del medico che ha dato le informazioni al paziente ……………………………….

DICHIARAZIONE DEL MEDICO CHE HA RACCOLTO IL CONSENSO

Io Sottoscritto (NOME-COGNOME)

nella mia qualità di
○ Sperimentatore Principale

○ Delegato dello Sperimentatore principale

DICHIARO

che il Paziente ha acconsentito spontaneamente alla sua partecipazione alla sperimentazione

Dichiaro inoltre di:
○ aver fornito al Paziente esaurienti spiegazioni in merito alle finalità della sperimentazione, alle procedure, ai possibili rischi e benefici e alle sue possibili alternative;

○ aver verificato che il Paziente abbia sufficientemente compreso le informazioni fornitegli;

○ aver lasciato al Paziente il tempo necessario e la possibilità di fare domande in merito alla Sperimentazione;

○ di aver illustrato chiaramente la possibilità di ritirarsi in qualsiasi momento dalla sperimentazione o di modificare le scelte fatte;

○ non aver esercitato alcuna coercizione od influenza indebita nella richiesta del presente consenso;

○ avere fornito al paziente informazioni su come i risultati della sperimentazione gli/le saranno resi noti.
____ ________

Luogo e data ____

____
Nome Cognome (stampatello) del medico che ha fornito le Informazioni e che ha raccolto il consenso

Firma (e timbro)

Il presente modulo e parte integrante e deve essere conservato insieme al modulo informativo per il consenso informato
