# Supplementary material for: Polygenic Risk Scores disclosure for cardiovascular prevention: Protocol of the Personalized HeartCare (PHC) trial
Source: PLoS One. 2026 Apr 6;21(4):e0345294. doi: 10.1371/journal.pone.0345294 (PMC13052841; doi:10.1371/journal.pone.0345294)
Supplement: S4 File — (PDF) [file pone.0345294.s004.pdf]

Questionnaire PHC

Questionnaire on Lifestyle, Family History, and Work History ………… 2

Section 1 – Lifestyle ……………………………………………………… 2

Section 2 – Family History ……………………………………………… 6

Section 3 – Laboratory Analysis ……………………………………… 8

Values and Preferences Questionnaire ………………………………………. 9

The Feelings About genomiC Testing Results (FACToR) Questionnaire.. 11

Feasibility Questionnaire on the Application of PRS …………………….. 12

Acceptability Questionnaire on the Application of PRS ….………………. 13

GAD-7 Anxiety Questionnaire ……………………………………………... 14

1. SECTION 1

1.1. PERSONAL DATA

- Gender: M / W
- Age: … years old
- Nationality: Italian or Other (if other, please specify) ……………….
- Municipality of:
- Residential Address:
- Enter your ZIP code:

1.2 What is your or degree of education?

- No degree
- Elementary schooling
- Middle school diploma
- High school diploma
- Bachelor's degree
- Master's degree
- PhD degree
- Don't know/Prefer not to answer

1.3 What is your marital status?

- Single
- Boyfriend/girlfriend
- Civil partner
- Married
- Separated/divorced
- Widow/widower
- Don't know/Prefer not to answer

1.4 Where does he live?

- In a large city (population more than 200,000)
- In a city (less than 200,000 inhabitants)
- In a rural area (e.g., countryside, mountains)
- Has your residence changed often in recent years.
- Don't know/Prefer not to answer

1.5 Do you have a job?

- Yes:

    - Full-time
    - Less than five days a week
    - Part-time
    - Other …….
    - What is your job role? (open-ended question)
    - What occupational risks are you exposed to? (open-ended question)
    - How long have you been doing this job? (open-ended question)
    - Did you have a different job before your current one?

      Yes ________________ No
    - If yes, what was your previous job role? (open-ended question)
    - What occupational risks were you exposed to in that job? (open-ended question)
- No:
    - Unemployed
    - Retired due to disability
    - Retired due to age
    - Homemaker
    - Other…..
    - If you worked previously, what was your job role? (open-ended question)
    - What occupational risks were you exposed to? (open-ended question)
- I don't know / I prefer not to answer

1.2 DIET

1. How many tablespoons of olive oil do you consume including both for dressing and cooking)?

Any number greater than or equal to 0 is allowed.

2. How many servings (a serving being approximately 200 grams) of vegetables do you consume **per day**?

Any number greater than or equal to 0 is allowed.

3. How many servings of fruit (a serving being approximately 150 grams, e.g.,: one apple) do you consume **per day**?

Any number greater than or equal to 0 is allowed.

4. How many servings of bread, pasta, rice or cereals do you consume **per day**? (For a serving of bread, it is 50 grams, for pasta or cereals, it's 80 grams)

Any number greater than or equal to 0 is allowed.

5. How many times **per day** do you consume sweets (e.g., cookies, pastries, slices of cake, croissants and brioches)?

Any number greater than or equal to 0 is allowed.

6 A: How many alcoholic units do you consume **per day**? (FOR WOMEN)

(An alcoholic unit corresponds to 12 grams of pure alcohol and is equivalent to:

- a glass of wine (125 ml at 12°)
- a can of beer (330 ml at 4.5°)
- an aperitif (80 ml at 38°)
- a shot of spirits (40 ml at 40°)

Any number greater than or equal to 0 is allowed.

6 B: How many alcoholic units do you consume per day? (FOR MEN)

(One alcoholic unit corresponds to 12 grams of pure alcohol and is equivalent to:

- a glass of wine (125 ml at 12°)
- a can of beer (330 ml at 4.5°)
- an aperitif (80 ml at 38°)
- a shot of spirits (40 ml at 40°)

Any number greater than or equal to 0 is allowed.

7. How many servings (a serving being approximately 80 grams) of broad leafy vegetables (such as spinach) do you consume **per week**?

Any number greater than or equal to 0 is allowed.

8. How many servings (a serving being approximately 80 grams) of berries do you consume **per week**?

Any number greater than or equal to 0 is allowed.

9. How many servings (a serving being approximately 100 grams) of red meat do you consume **per week**?

Any number greater than or equal to 0 is allowed.

10. How many servings (a serving being approximately 150 grams) of fish (not fried) do you consume in **per week?**

Any number greater than or equal to 0 is allowed.

11. How many servings (a serving being approximately 100 grams) of chicken (not fried) do you consume **per week?**

Any number greater than or equal to 0 is allowed.

12. How many servings of cheese (a serving being at least 50 grams) do you consume **per week?**

Any number greater than or equal to 0 is allowed.

13. How many servings (a serving being approximately 10 grams) of butter do you consume **per week**?

Any number greater than or equal to 0 is allowed.

14. How many servings of fresh or canned legumes (approximately 150 grams) or dried legumes (approximately 50 grams) legumes do you consume **per week**?

Any number greater than or equal to 0 is allowed.

15. How many servings (a serving being approximately 30 grams) of dried fruits (e.g., almonds, hazelnuts, walnuts) do you consume **per week**?

Any number greater than or equal to 0 is allowed.

16. How many meals do you consume in fast food restaurants (example: McDonald's or Burger King) **per week**?

Any number greater than or equal to 0 is allowed.

1.3 PHYSICAL ACTIVITY

1a. How many minutes of intense intensity physical activity (example: e.g., lifting weights, heavy yard work, aerobic activities such as running or bicycling at high speed) do you perform per week?

Any number greater than or equal to 0 is allowed. (in minutes)

1b. How many minutes of moderate-intensity physical activity (example: e.g., carrying light weights, bicycle rides at a regular speed, gym activities, garden work, prolonged physical work at home, walking at a brisk pace) do you do per week?

Any number greater than or equal to 0 is allowed. (in minutes)

NB: for the final score the two questions will be merged.

1.4 NICOTINE EXPOSURE

1. Are you a smoker?

-Never been a smoker

-I am currently a smoker (if yes, please answer in the box below):

- **how many cigarettes per day?** (a number greater than 0)
- **for how many years?** (a number greater than 0)

-Former smoker: (if yes, open the boxes below)

- Less than one year ago or currently using (**open the box below**):
    - Heated tobacco cigarettes (Iqos, Glo etc.).
    - electronic cigarettes
    - Other (please specify)
- -from 1 to 5 years
- - more than five years ago

1.5 SLEEPING TIME

1. On average, how many hours do you sleep in a 24-hour period?

Any number greater than or equal to 0 (in hours) is allowed.

2. SECTION 2

2.1 PERSONAL HISTORY

1. How many liters of water can you drink in a day?

-Less than half a liter

-A liter

-One and a half liters

-Two liters

-Two and a half liters

2. How often do you add salt to your food at the table?

-Never or rarely

-Rather often

-Always or very often

3. Do you find yourself very thirsty, particularly after a meal?

-Never or rarely

-Rather often

-Always or very often

4. Do you consume medication on a daily basis?

-Yes

-No

If yes please indicate which ones (open answer)

-Anxiolytics

-Antihypertensives

-Anti-inflammatories

-Antihypertensives

-Hypocholesterolemic

-Other (indicate which)

FOR WOMEN ONLY

Menopause:

-Yes

-No

-Don't know

2.2 FAMILY BACKGROUND

Among your family members (parents, siblings, children) has anyone presented with one or more of the following illnesses?

5. Early cardiovascular disease (heart attack, angina, cardiac ischemia, sudden cardiac death, angioplasty (balloon/stent) or coronary artery bypass), respond:

-No

-Yes

-Don't know

6. Early cerebrovascular disease (stroke, hemorrhagic stroke, cerebral ischemia, transient cerebral ischemia (also called TIA), respond:

-No

-Yes

-Don't know

Also among his family members, someone introduced:

7. High cholesterol (in parents, brothers, sisters, children)

-No

-Yes

-Don't know

8. Diabetes (in at least one of the birth parents)

-No

-Yes

-Don't know

9. Diabetes (in brothers, sisters, children)

-No

-Yes

-Don't know

10. Diabetes (in grandparents, uncles, cousins)

-No

-Yes

-Don't know

11. Hypertension (father)

-No

-Yes

-Don't know

12. Hypertension (mother)

-No

-Yes

-Don't know

3. SECTION 3

3.1 BLOOD PRESSURE (in mmHg)

Systolic blood pressure:

Diastolic blood pressure:

If you take blood pressure lowering medications put yes or no.

(If yes, specify which ones, open answer)

3.2 CHOLESTEROL (in mg/dL)

Non-HDL cholesterol (non-high-density cholesterol, which comprises the sum of total cholesterol minus HDL cholesterol):

If you take cholesterol-lowering medications, please answer yes or no. If yes, specify which ones (open-ended response).

LAST EXAM DATE (if under six months) (open-ended section)

3.3 GLICEMIA (mg/dL) or HbA1c (%)

If you have diabetes say yes or no.

Fasting blood glucose:

Glycated hemoglobin HbA1c:

LAST EXAM DATE (if under six months) (open-ended section)

3.4 BODY MASS INDEX

Height: in cm

Weight: in kg

Based on these values the BMI will be automatically calculated

VALUES AND PREFERENCES QUESTIONNAIRE

HBCVD

(Tovar EG, Rayens MK, Clark M, Nguyen H. Development and psychometric testing of the Health Beliefs Related to Cardiovascular Disease Scale: preliminary findings. J Adv Nurs. 2010 Dec;66(12):2772-84. doi: 10.1111/j.1365-2648.2010.05443.x. Epub 2010 Sep 10. PMID: 20831570.)

The response format for each item is a 4-point Likert scale

1 = Strongly Disagree; 2 = Disagree; 3 = Agree; 4 = Strongly Agree

1. It is likely that I will suffer from a heart attack or stroke in the future
2. My chances of suffering from a heart attack/stroke in the next few years are great
3. I feel I will have a heart attack or stroke sometime during my life
4. Having a heart attack or stroke is currently a possibility for me
5. I am concerned about the likelihood of having a heart attack/stroke in the near future
6. Having a heart attack or stroke is always fatal
7. Having a heart attack or stroke will threaten my relationship with my significant other
8. My whole life would change if I had a heart attack or stroke
9. Having a heart attack or stroke would have a very bad effect on my sex life
10. If I have a heart attack or stroke I will die within 10 years
11. Increasing my exercise will decrease my chances of having a heart attack or stroke Be
12. Eating a healthy diet will decrease my chance of having a heart attack or stroke
13. Eating a healthy diet and exercising for 30 minutes most days ... to prevent a heart attack/stroke
14. When I exercise I am doing something good for myself
15. When I eat healthy I am doing something good for myself
16. Eating a healthy diet will decrease my chances of dying from cardiovascular disease
17. I don’t know appropriate exercises to perform to reduce my risk of developing cardiovascular disease
18. It is painful for me to walk for more than 5 minutes
19. I have access to exercise facilities and/or equipment
20. I have someone who will exercise with me
21. I don’t have time to exercise for 30 minutes a day on most days of the week
22. I don’t know what is considered a healthy diet that would prevent me from developing cardiovascular disease

23. I don’t have time to cook meals for myself
24. I cannot afford to buy healthy foods
25. I have other problems more important that worrying about diet and exercise

The Vanderbilt PRS-KS

(Stubbs D, Hooker G, Li Y, Richter L, Bick A, Development and Validation of the Vanderbilt PRS-KS, an Instrument to Quantify Polygenic Risk Score Knowledge, Genetics in Medicine Open (2023), doi: https://doi.org/10.1016/j.gimo.2023.100822.)

1. Polygenic risk scores are based on genetic changes in more than one gene
2. All people who receive a high-risk result on a polygenic risk score for a disease will develop that disease.
3. A polygenic risk score can combine other health determinants beyond genetics.
4. Polygenic risk scores have the same accuracy regardless of the disease being testing for.
5. Polygenic risk scores have the same accuracy for all people, regardless of their racial or ethnic background.
6. If you receive a high polygenic risk score result, your children will have a high polygenic risk result.
7. Your polygenic risk score could find a decreased risk for disease.

The Feelings About genomiC Testing Results (FACToR) questionnaire

The following questions concern how you have felt after receiving your genetic test results. Please indicate how much you have experienced each specific feeling during the past week by circling only one answer for each question:

0 = not at all, 1 = a little, 2 = moderately, 3 = quite a bit, or 4 = extremely.

1. In the past week:

How upset did you feel about your genetic test result?

2. In the past week:

How happy did you feel about your genetic test result?

3. In the past week:

How anxious or nervous did you feel about your genetic test result?

4. In the past week:

How relieved did you feel about your genetic test result?

5. In the past week:

How sad did you feel about your genetic test result?

6. In the past week:

How frustrated did you feel that there are no definite disease prevention guidelines for you?

7. In the past week:

How uncertain did you feel about what your genetic test result means for you?

8. In the past week:

How uncertain did you feel about what your genetic test result means for your child(ren) and/or family's risk of disease?

9. In the past week:

How much did you feel that you understood clearly your choices for disease prevention or early detection?

10. In the past week:

How concerned did you feel that your genetic test result would affect your health insurance status?

11. In the past week:

How helpful was the information you received from your genetic test result in planning for the future?

12. In the past week:

How concerned did you feel that your genetic test result would affect your employment status?

Feasibility Questionnaire on the Application of PRS

Patient Section

For each question, please indicate how much you agree by selecting a number from 1 to 4:

1 = Strongly disagree, 2 = Somewhat disagree, 3 = Somewhat agree, 4 = Strongly agree

1. Knowing the results of my Polygenic Risk Score has made me more proactive in my efforts to preserve my health.
2. Understanding the result of the Polygenic Risk Score was easy.
3. Knowing the result of the Polygenic Risk Score caused me frustration.
4. Knowing the result of the Polygenic Risk Score has improved my quality of life.
5. I would recommend others to undergo Polygenic Risk Score testing.
6. Knowing the result of the Polygenic Risk Score has motivated me to improve my lifestyle.
7. I believe that by participating in the study, my lifestyle has become healthier.

Clinician Section

For each question, please indicate how much you agree by selecting a number from 1 to 4:

1 = Strongly disagree, 2 = Somewhat disagree, 3 = Somewhat agree, 4 = Strongly agree

1. The new procedures introduced by the study (Polygenic Risk Score) could become part of my daily clinical practice.
2. The activities required by the study created difficulties in the routine workflow of my clinical practice.
3. The time and effort required for the study activities were acceptable.
4. I found it difficult to explain the results of the Polygenic Risk Score analysis to the patient.

Acceptability Questionnaire on the Application of Polygenic Risk Scores (PRS) for the clinicians

Please indicate how confident you would feel in each of the following situations:

0 = not at all, 1 = a little, 2 = moderately, 4 = extremely.

1. Talking with a patient about polygenic risk score (PRS) testing for disease
2. Recommending a polygenic risk score test to a patient with a family history of disease
3. Answering patients' questions about polygenic risk score testing
4. Interpreting the results of a polygenic risk score test
5. Explaining lifetime disease risk to a patient based on the result of a polygenic risk score test
6. Managing a patient with an increased polygenic risk of disease
7. Managing a patient with a reduced polygenic risk of disease

GAD-7 Anxiety

Over the last two weeks, how often have you been bothered by the following problems?

0=Not at all 1=Several days 2=More than half the days 3=Nearly every day

1. Feeling nervous, anxious, or on edge
2. Not being able to stop or control worrying
3. Worrying too much about different things
4. Trouble relaxing
5. Being so restless that it is hard to sit still
6. Becoming easily annoyed or irritable
7. Feeling afraid, as if something awful might happen
